# Acceptance in Patients With Cancer: A Scoping Review

**DOI:** 10.1002/cnr2.70324

**Published:** 2025-10-30

**Authors:** Joost Dekker, Chris Welling, Mariette Labots

**Affiliations:** ^1^ Department of Psychiatry Amsterdam University Medical Center, Vrije Universiteit Amsterdam Amsterdam the Netherlands; ^2^ Amsterdam Public Health Mental Health Program Amsterdam the Netherlands; ^3^ Cancer Centre Amsterdam Cancer Treatment and Quality of Life Amsterdam the Netherlands; ^4^ Department of Clinical Psychology Vrije Universiteit Amsterdam Amsterdam the Netherlands; ^5^ Department of Medical Oncology Amsterdam University Medical Center, Vrije Universiteit Amsterdam Amsterdam the Netherlands

**Keywords:** acceptance, cancer, definition, review, stress

## Abstract

**Background:**

Cancer is generally perceived as a major stressor. Occasionally, patients are able to accept their diagnosis and prognosis, and it is not uncommon for patients who initially experience great distress to eventually learn to accept their disease. A deeper understanding of acceptance can enable clinicians to better support patients in achieving a more peaceful state of mind. The purpose of this literature review was to provide a comprehensive overview of existing research on acceptance in patients with cancer.

**Recent Findings:**

Study selection resulted in the inclusion of 54 studies. All studies except one were published after the year 2000. Acceptance was defined in terms of cognition, emotion, behavior, spiritual processes, social processes, or other terms. Acceptance focused on disease, illness, cancer, poor prognosis/imminent death, or was not specified, and was measured by interview or questionnaire. Evidence was found for a range of factors associated with acceptance.

**Conclusions:**

Acceptance is a relatively new field of research, characterized by significant heterogeneity in both its definition and focus, as well as the hypothesized determinants and outcomes. To advance this field, it is essential to develop a generally accepted definition of acceptance and to consistently specify its focus. The present scoping review provides a solid foundation for this endeavor.

## 1 Introduction

Cancer and its treatment are generally experienced as a major stressor, leading to distress. The reported prevalence of general distress ranges from 35% to 52% [1, 2]. Distress may stem from uncertainty about prognosis, the possibility of a fatal outcome, interference with life goals, symptoms such as pain, fatigue, or nausea, problems in the emotional, social, occupational, or spiritual domain, and practical difficulties [3, 4, 5]. Distress is undesirable in itself and a risk factor for poor decision making in clinical encounters [6], nonadherence to medical treatment [7], and a poor outcome in the physical, mental, and social life domain [8].

According to our clinical experience, distress does not affect all patients with cancer. Occasionally, patients are able to accept their diagnosis and prognosis, even those with advanced stages of the disease and despite the likelihood of a fatal outcome based on their staging. Instead of reacting with distress, these patients respond in a peaceful manner, showing signs of acceptance. In addition, it is not uncommon for patients who initially experience great distress to eventually learn to accept their disease, developing an accepting attitude over time.

Research on acceptance in patients with cancer has been earlier reviewed. Secinti et al. [9] demonstrated negative associations between acceptance of cancer and general distress, cancer‐specific distress, depressive symptoms, and anxiety symptoms. Quinto et al. [10] reported positive associations between meaning in life and the acceptance of cancer. Recently, Fawson et al. [11] reviewed the literature on associations between processes related to acceptance and commitment therapy and distress in patients with cancer. They reported that higher scores on flexible processes (acceptance, present moment awareness, self‐compassion) were associated with lower distress, while higher scores on inflexible processes (experiential avoidance, cognitive fusion) were associated with higher distress.

While these reviews provide important insights, to the authors' knowledge no comprehensive overview of studies on acceptance in patients with cancer exists. A deeper understanding of acceptance can enable clinicians to better support patients experiencing cancer‐related distress in achieving a more accepting state of mind. The purpose of this literature review was therefore to provide a comprehensive overview of existing research on acceptance in patients with cancer.

## 2 Materials and Methods

A protocol for the scoping review was developed, using the five‐stages framework from Arksey and O'Malley [12]. The five stages included: (i) identification of the research question; (ii) identifying relevant studies; (iii) study selection; (iv) charting the data; and (v) collating, summarizing, and reporting the results [12]. The results are reported in accordance with the PRISMA guidelines for reporting scoping reviews [13]. No ethics approval was required for this review.

### 2.1 Identification of the Research Question

We developed the following research question: What is known from the existing literature on acceptance in patients with cancer?

### 2.2 Identification of Relevant Studies

Potentially relevant studies were identified through a systematic search using a combination of operator‐specific keywords in PubMed and PsycInfo. The search terms focused on cancer and acceptance. See Appendix A for the search strategy in PubMed. References of included reviews were hand‐searched for eligible studies. The literature search was conducted on 15 November 2023 and updated on 3 January 2025.

### 2.3 Study Selection

Studies were selected if they (i) included a sample of patients with cancer or cancer survivors, aged 18 years or older; (ii) reported data on accepting or dealing in a peaceful way with the disease or one's fate; (iii) were published in English; (iv) were published in peer‐reviewed journals; and (v) had the full text available. Abstracts and articles were excluded if they did not include empirical data.

The study selection consisted of two stages. In the first stage, one reviewer (C.W.) screened all studies by title and abstract. Inter‐rater reliability was checked in a random sample of 61 articles (10% of the initially identified articles). Two reviewers (C.W. and J.D.) independently selected articles. Kappa was calculated, with *κ* = 0.80 as the preset threshold value that was to be met. The observed *κ* was 0.89. Because of the high interrater agreement, one reviewer (C.W.) performed the final study selection. In the second stage, the full texts of all studies identified as potentially relevant were read by one reviewer (C.W.). In case of uncertainty, the selection was discussed with two other reviewers (J.D. and M.L.).

### 2.4 Charting the Data

The following information was extracted from the included studies: authors, year of publication, title of article, country, aim of the study, design, sample size, cancer type (by organ/system, e.g., breast, lung, gastrointestinal), cancer stage, time since diagnosis, age, gender, definition of acceptance, focus of acceptance, measurement of acceptance, acceptance assessed at a specific point in care (diagnosis, test results, etc.), data on acceptance (categorized as a group average or the number of accepting patients), factors (presumably) influencing acceptance, association with metastatic disease, association of acceptance with quality of life and functioning, and acceptance as mediator between other constructs. One reviewer (C.W.) Extracted the data and the second reviewer (J.D.) checked accuracy

## 3 Results

### 3.1 Study Selection

**FIGURE 1:**
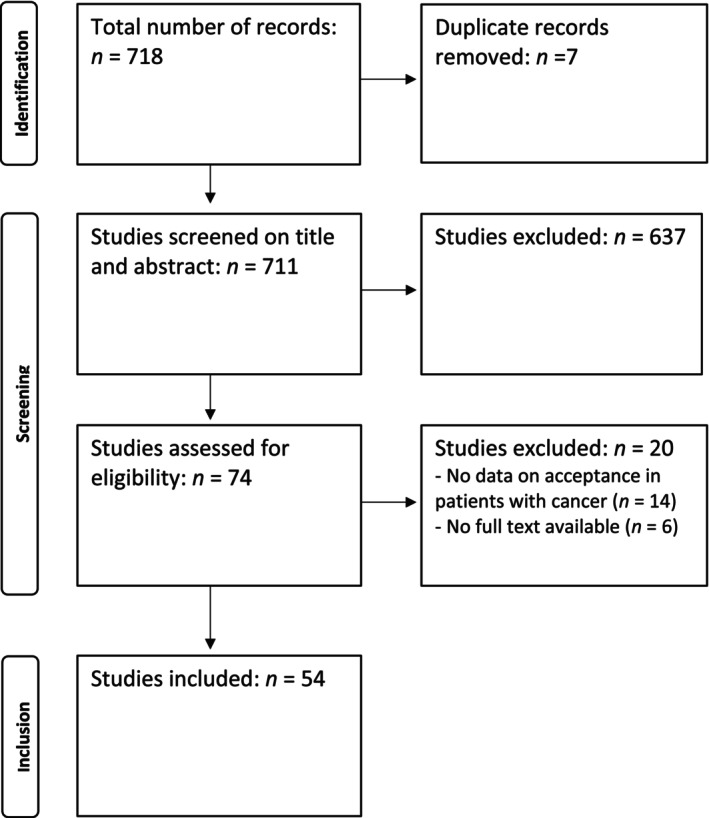
Selection of studies.

The flow diagram and reasons for exclusion are shown in Figure 1. The initial search yielded 718 records. Upon removing duplicates, 711 unique studies remained. Screening on title and abstract excluded 637 studies. Seventy‐four studies were screened based on their full text, leading to the inclusion of 54 studies. The full information extracted from the studies is displayed in Appendix B, Table B1 and summarized in Tables 1, 2, 3.

**TABLE 1:** Characteristics of included studies.

Characteristic	References
Year of publication	
Before 2000	[14]
2000 and later	[9, 10, 11, 15, 16, 17, 18, 19, 20, 21, 22, 23, 24, 25, 26, 27, 28, 29, 30, 31, 32, 33, 34, 35, 36, 37, 38, 39, 40, 41, 42, 43, 44, 45, 46, 47, 48, 49, 50, 51, 52, 53, 54, 55, 56, 57, 58, 59, 60, 61, 62, 63, 64]
Country	
Europe	[10, 11, 14, 15, 16, 17, 18, 19, 21, 22, 23, 26, 27, 28, 31, 33, 35, 36, 37, 38, 42, 43, 44, 45, 47, 48, 49, 55, 56, 58, 59, 60, 62]
North America	[9, 20, 24, 25, 30, 39, 40, 41, 50, 51, 53]
Asia	[29, 34, 46, 52, 54, 57, 61, 63, 64]
Africa	[32]
Cancer	
One type of cancer	[15, 18, 19, 22, 23, 26, 28, 30, 31, 32, 35, 36, 37, 45, 47, 49, 54, 57, 58, 59, 60, 61, 63, 64]
Multiple types of cancer	[9, 10, 11, 14, 16, 17, 21, 24, 25, 27, 29, 33, 34, 38, 40, 41, 43, 44, 48, 50, 51, 52, 53, 55, 56, 62]
Not reported	[20, 39, 42, 46]
Sample size	
< 100	[14, 18, 21, 25, 26, 30, 42, 46, 47, 50, 55, 57, 61]
100–500	[15, 16, 19, 20, 22, 23, 24, 27, 28, 29, 31, 32, 34, 35, 36, 37, 39, 40, 43, 44, 45, 49, 51, 52, 53, 54, 56, 58, 59, 60, 62, 63, 64]
> 500	[17, 33, 38, 41, 48]
Review	[9, 10, 11]
Sex	
Female	[10, 15, 16, 23, 26, 30, 31, 36, 37, 40, 55, 57, 58, 59, 61, 63, 64]
Male	[22, 35]
Mixed	[9, 11, 14, 17, 18, 19, 20, 21, 24, 25, 28, 29, 32, 33, 34, 38, 39, 41, 42, 43, 44, 45, 46, 47, 50, 51, 52, 53, 54, 56, 60, 62]
Not reported	[27, 48, 49]
Design	
Cross‐sectional	[15, 16, 17, 19, 20, 21, 22, 23, 24, 25, 26, 27, 28, 29, 30, 31, 32, 33, 34, 35, 36, 38, 39, 40, 41, 42, 45, 46, 47, 48, 49, 50, 51, 53, 54, 55, 56, 57, 58, 59, 60, 61, 62, 63, 64]
Longitudinal	[14, 18, 37, 43, 44, 52]
Review	[9, 10, 11]

**TABLE 2:** Characterization of acceptance.

Aspect of acceptance	References
Acceptance defined in terms of^a^	
Cognition	[9, 10, 11, 14, 19, 24, 25, 32, 34, 38, 42, 43, 44, 46, 48, 50, 51, 56, 61, 62, 63, 64]
Emotion	[14, 17, 20, 30, 40, 43, 46, 48, 51, 52, 57, 61, 64]
Behavior	[9, 10, 14, 25, 32, 38, 46, 48, 51, 56, 57, 61, 62, 63, 64]
Spiritual processes	[14, 41]
Social processes	[14]
Other	[31, 36, 37, 54]
No definition	[15, 16, 18, 21, 22, 23, 26, 27, 28, 29, 33, 35, 45, 47, 49, 53, 55, 58, 59, 60]
Focus of acceptance^a^	
Illness, disease, health, pain	[9, 10, 11, 15, 16, 17, 18, 19, 21, 22, 23, 25, 27, 28, 31, 33, 35, 36, 37, 41, 42, 45, 47, 48, 49, 54, 55, 56, 57, 58, 59, 60, 63]
Cancer	[9, 24, 25, 29, 30, 51, 61, 62]
Poor prognosis/death	[14, 25, 34, 43, 44, 46, 50, 52]
Self, emotions	[11]
Not specified	[9, 10, 11, 20, 24, 26, 29, 30, 32, 33, 38, 39, 40, 41, 51, 53, 64]
Measurement of acceptance^a^	
Interview	[14, 25, 26, 46, 50, 57, 61]
Acceptance of Illness Scale	[10, 15, 16, 17, 18, 19, 21, 22, 23, 27, 28, 31, 35, 42, 45, 47, 48, 49, 54, 55, 56, 60, 63]
Fetzer multidimensional measure of religiousness/spirituality	[20]
Peace, Equanimity, and Acceptance in the Cancer Experience (PEACE) Scale	[24, 29, 30, 39, 51, 62]
Brief Cope/COPE questionnaire	[9, 10, 32, 40]
Functional Assessment of Chronic Illness‐Spiritual Well‐Being (FACIT‐Sp) scale	[33, 41]
Buddhist Death Acceptance Scale	[34]
Acceptance of Life with the Disease Scale	[36, 37, 58, 59]
Acceptance and Action Questionnaire	[38]
Life Attitude Profile‐Revised	[43]
Five item Likert scale	[44]
Acceptance of Disability Scale‐Revised	[10]
Cancer Behavior Inventory	[9]
Illness Cognitions Questionnaire	[9]
Single item on acceptance	[52, 53]
Positive Acceptance Scale	[64]
Acceptance assessed at a specific point in treatment	
Life expectancy < 12 months	[14, 39, 50, 52]
Transition in treatment	[18, 33, 35, 37, 38, 44]
Not, or not specified	[9, 10, 15, 16, 17, 19, 20, 21, 22, 23, 24, 25, 26, 27, 28, 29, 30, 31, 32, 34, 36, 40, 41, 42, 43, 45, 46, 47, 48, 49, 51, 53, 54, 55, 56, 57, 58, 59, 60, 61, 62, 63, 64]
Data on acceptance, reported as^a^	
Group average	[15, 16, 17, 18, 19, 21, 22, 24, 27, 28, 29, 30, 31, 32, 34, 35, 36, 37, 38, 41, 42, 43, 44, 45, 47, 48, 49, 51, 54, 55, 56, 58, 63, 64]
Number of accepting patients	[14, 20, 26, 32, 52, 53]
Qualitative description	[46, 50, 57]
Not reported	[9, 10, 11, 23, 25, 33, 39, 40, 59, 61, 62]

^a^ More than one category may apply per study.

**TABLE 3:** Variables associated with acceptance.

Variables associated with acceptance	References
Factors influencing acceptance (according to the authors' conceptualization)^a^	
Tumor type	[16, 29, 43, 45, 48]
Metastatic disease	[9, 16, 17, 21, 34, 43, 63]
Treatment and care	[17, 20, 23, 39, 48, 50, 57, 60, 61, 63]
Time since diagnosis, time to death	[29, 43, 52]
Symptoms	[9, 24, 29, 37, 39, 44, 45, 56]
Psychological and existential factors	[10, 25, 36, 39, 42, 44, 53, 55, 58, 59, 60, 61, 63, 64]
Quality of life	[14]
Sociodemographic factors	[14, 22, 31, 34, 35, 39, 43, 44, 48, 49, 53, 60, 62, 63]
Partner, significant others	[14, 15, 17, 18, 25, 36, 42, 59, 61]
Association of acceptance with quality of life and functioning^a^	
Psychological and spiritual outcomes, coping, symptoms, physical functioning, social functioning, spiritual outcomes, and quality of life	[9, 11, 20, 23, 26, 28, 30, 32, 35, 38, 43, 45, 50, 52, 53, 56, 62]
Advance care planning	[20]
Quality of death	[20]
Acceptance as a mediator between	
Symptoms and psychological distress	[29]
Benefit finding and depression	[40]
Relinquishing control, coping efficacy and quality of life	[41]
Perceived injustice and psycho‐spiritual outcomes	[51]
Neuroticism/extraversion and depression	[54]

^a^ More than one category may apply per study.

### 3.2 Study Characteristics

All studies except one were published after the year 2000 (see Table 1). Most of the 54 studies originated from Europe (*n* = 33), of which 24 were from Poland. The remaining studies were from North America (*n* = 11), Asia (*n* = 9) and Africa (*n* = 1).

The studies aimed to describe the process of acceptance, to develop a measure of acceptance, to assess the level of acceptance, factors and processes associated with and leading to acceptance, the association of acceptance with symptoms, quality of life, psychological functioning and behavior, and the mediating role of acceptance in other psychological processes.

Studies included patients with one type (*n* = 24) or multiple types of cancer (*n* = 26) (not reported *n* = 4). These included breast, prostate, gastrointestinal, lung, gynecological, hematologic, or other types of cancer. Cancer stage was described in terms such as palliative, advanced stage, metastatic, not metastasized, early stage, operated, cancer recurrence, or using specific tumor stages. Information on the time since diagnosis was available in 25 studies. The sample size was < 100 in 13 studies, from 100 to 500 in 33 studies, and > 500 in 5 studies, ranging from 13 to 1187; reviews included 464, 15 488, and 17 195 patients, respectively. Patients' age was reported in 46 studies. Most studies (*n* = 33) included both male and female patients; 19 studies included only male or female patients (three studies did not report on sex distribution). The studies had a cross‐sectional (*n* = 45) or longitudinal (*n* = 6) design or were a systematic review or meta‐analysis (*n* = 3).

### 3.3 Acceptance

Acceptance was *defined* in terms of cognition (e.g., acknowledging the reality of the illness, full awareness of experiences), emotion (e.g., emotional experience of coming to accept one's terminal illness, absence of fear, sense of peace), behavior (e.g., a person does not attempt to change the course of their illness), spiritual processes (e.g., faith and spiritual values, religious and spiritual processes), social processes (e.g., sharing) or other terms (e.g., coming to terms with own state of health) (see Table 2) Acceptance *focused* on illness, disease, or health (e.g., feeling unnecessary because of the disease, accepting pain), cancer (e.g., accepting the cancer diagnosis), poor prognosis/imminent death (e.g., knowledge that disease cannot be cured and patient will likely die in the near future), self and emotions, or was not specified (e.g., the extent of feeling a sense of inner calm and tranquility, accepting the situation as it is). Acceptance was *measured* through interview or with one of the many questionnaires available (see Table 2). In Appendix B, Table B2 we have listed the definition, focus, and measurement of acceptance in studies that provided a definition for a better overview of the conceptualization of acceptance. Acceptance was *assessed at* < 12 months life expectancy, at a treatment transition (e.g., after surgery), and in most studies at an unspecified moment during or after treatment. Most studies reported *data* on acceptance as a group average (e.g., the mean on a questionnaire); other studies reported the number of patients categorized as accepting, gave a qualitative description of acceptance, or did not report data on acceptance (e.g., a study on acceptance as a mediator, without reporting the acceptance scores).

### 3.4 Factors Presumably Influencing Acceptance

Factors that influence acceptance—according to the authors' conceptualization—included type of tumor, metastatic disease (e.g., associated with lower acceptance), treatment and care (e.g., chemotherapy associated with lower acceptance), time since diagnosis, symptoms (e.g., more symptoms associated with less acceptance), psychological and existential factors (e.g., letting go of control, mindfulness), sociodemographic factors (e.g., sex, socioeconomic status), and the partner (e.g., accepting attitude of the partner, quality of communication) (see Table 3).

### 3.5 Association of Acceptance With Quality of Life and Functioning

Acceptance was found to be associated with psychological and spiritual outcomes (e.g., less distress, more hope for connection with others), coping (e.g., a constructive way of coping with the disease), symptoms (e.g., less pain), physical functioning, social functioning, quality of life, advance care planning, and quality of death (see Table 3).

### 3.6 Acceptance as a Mediator

Acceptance was found to mediate the relationship between various constructs (e.g., acceptance mediated the relationship between symptoms and psychological distress). See Table 3.

## 4 Discussion

The definition of acceptance in patients with cancer is not agreed upon: our review revealed that acceptance has been conceptualized in vastly different ways. Furthermore, the review documented significant variations in the focus of acceptance, with some studies emphasizing acceptance of illness or disease (including cancer), others focusing on poor prognosis or imminent death, or referring to a general state of mental tranquility. In a relatively new field of research, with nearly all studies published post‐2000, it is understandable that there is no consensus on the definition and focus of acceptance. Nevertheless, such major discrepancies in both definition and focus preclude synthesizing findings and drawing overall conclusions. To advance the field, it is crucial to develop a generally accepted definition of acceptance and to consistently clarify its specific focus in each study.

**FIGURE 2:**
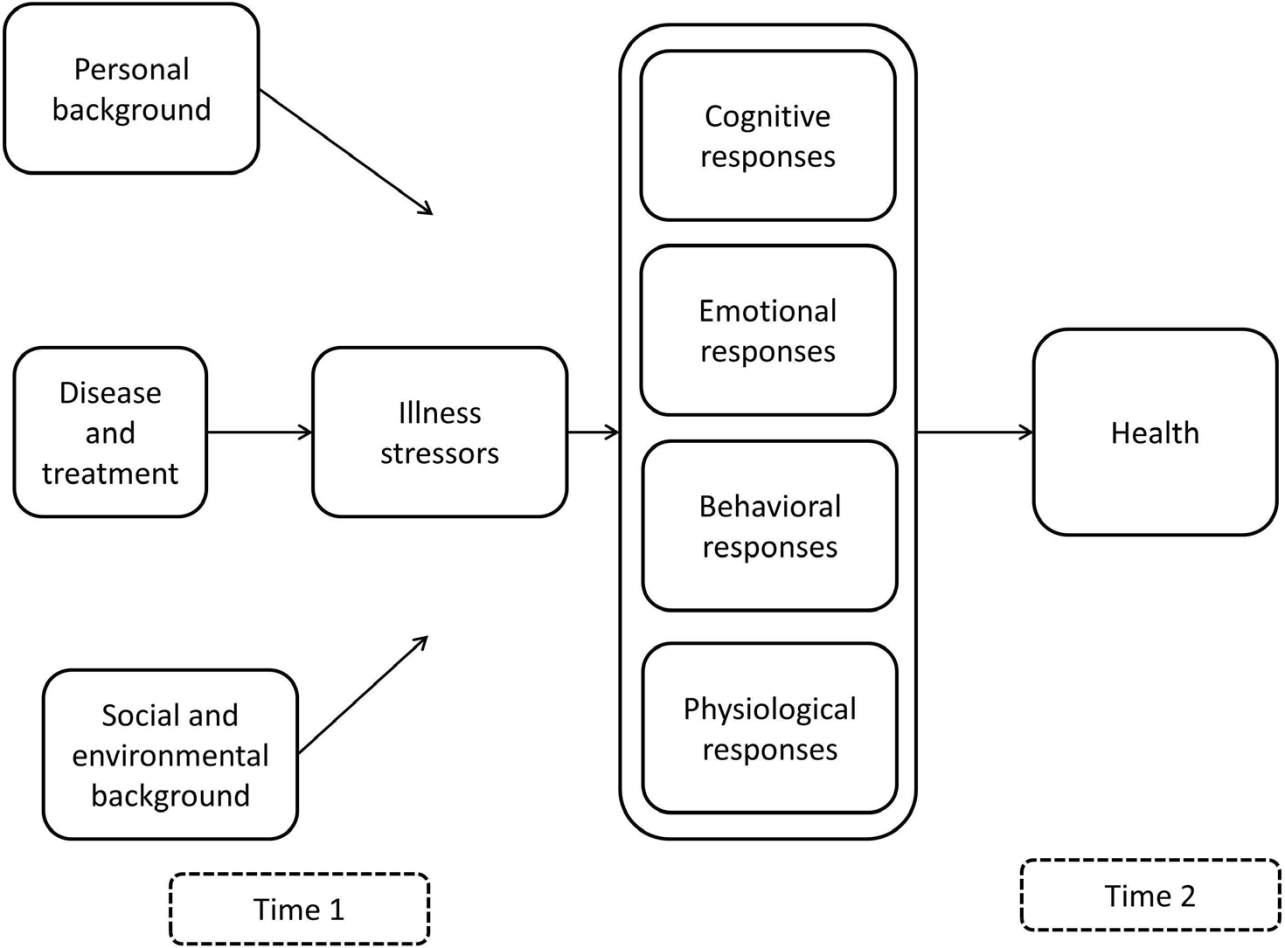
The general model on adjustment to disease and treatment [68].

The literature includes theoretical analyses of acceptance in general [65], in chronic disease [66], or in cancer [9], as well as syntheses of qualitative studies on acceptance in a specific disease [67]. This body of work provides valuable background knowledge for developing a comprehensive definition of acceptance. In addition, we recommend using the recently developed general model of adjustment to disease when formulating the definition of acceptance in patients with cancer (see Figure 2, [68]). This model was developed to describe how patients adjust to disease and treatment, and it can be used to characterize the process of acceptance. According to this model, cancer and its associated treatment are experienced on a continuum, ranging from a calm, accepting response to a major stressor. A calm, accepting experience triggers corresponding cognitive, emotional, behavioral, and physiological responses, which together lead to the experience of acceptance. When cancer and its treatment are perceived as stressors, corresponding cognitive, emotional, behavioral, and physiological responses emerge. However, over time, patients may learn to modify these responses to such an extent that acceptance can still be achieved. The patient's social and personal background (e.g., social support, spirituality) may modify these processes. We suggest using this model to define acceptance in terms of cognition (both the primary cognitive appraisal of cancer and its treatment, and the secondary cognitive appraisal of illness‐related stressors [69]), emotion, behavior, and physiology, as well as spirituality and social factors.

We further recommend to consistently specify the focus of acceptance. Without an explicit statement of the focus, patients may interpret questions on acceptance in various ways, leading to studies on “acceptance” that actually address different aspects of acceptance (e.g., acceptance of a poor prognosis versus acceptance of bodily disfigurement). We believe acceptance can not be focused on the disease, cancer, or treatment itself. Instead, acceptance can focus only on the subjectively experienced disease stressors induced by cancer and treatment (see Figure 2). Illness stressors, as listed in guidelines on distress management, include physical, emotional, social, practical, and spiritual concerns [3]. We propose to add cognitive concerns such as uncertainty about prognosis [5], the possibility of a fatal outcome, as well as life goal interference [4]. Developing a classification of cancer‐related illness stressors may help to clarify the focus of acceptance and make studies more comparable.

Various interviews and questionnaires were used to measure acceptance. Clearly, standardization of measurement is highly desirable to enhance the comparability of research results. This involves the development of both a standardized interview and a standardized questionnaire. These tools should be aligned well with the definition of acceptance that is to be established. Furthermore, criteria must be defined to interpret measurement results (does the score indicate a high or low degree of acceptance?).

For several factors, evidence was reported that they are associated with, and presumably influence, acceptance—referred to as hypothesized determinants of acceptance. These factors included disease‐ and treatment‐related variables, time since diagnosis, symptoms, psychological and existential factors, and sociodemographic and social factors. Our aim was not to provide a systematic review or meta‐analysis, where available evidence is carefully analyzed and weighed to determine whether a particular association has been demonstrated. Rather, our review highlights which factors have been studied, serving as a first step toward a systematic review and meta‐analysis. Future work is needed to synthesize the evidence, while accounting for the aforementioned heterogeneity in the definition and focus of acceptance, as well as the variation in study design. Notably, most studies employed a cross‐sectional design, precluding causal inference, with only a few longitudinal studies available. Finally, we suggest that the general model on adjustment to disease and treatment (see Figure 2) can guide the systematic development of research questions for systematic reviews.

A similar comment applies to the association between acceptance and quality of life and functioning, as the hypothesized outcomes of acceptance. Acceptance was found to be associated with psychological and spiritual outcomes, coping, symptoms, physical functioning, social functioning, quality of life, advance care planning, and quality of death. Future reviews and meta‐analyses are needed to determine what evidence exists for the hypothesized outcomes of acceptance. In addition, the conceptualization of the outcome of acceptance must be improved because studies were highly heterogeneous in this respect as well. In line with the general model on adjustment to disease and treatment [68], we propose to conceptualize the outcome in terms of the World Health Organization health domains: disorder or disease, somatic and mental functions and structures, activities and participation, social and environmental factors, and personal factors [70, 71]; to this could be added advance care planning, quality of death, and related constructs.

Evidence was reported on acceptance as a mediator between various constructs, which we consider building blocks for a future theoretical model on the role of acceptance in quality of life and death in patients with cancer. There is a need for further original research in this area, as only five studies examined acceptance as a mediator.

The present review is limited to studies published in English and in peer‐reviewed journals. We did not consider studies written in another language, nor did we examine the gray literature. However, we believe our conclusion on heterogeneity in both definition and focus and our conclusion on the assumed determinants and outcomes of acceptance would not change if we had. Another limitation is that we did not differentiate by stage of cancer, and in particular, we did not differentiate between patients and survivors. Cancer stage was reported in a variety of ways, precluding distinction between patients and survivors.

The clinical experience that some patients accept a poor prognosis in a peaceful way was the starting point for us to conduct this review. In the review on acceptance, we came across the great heterogeneity in the focus of acceptance. This was an impediment to making generalizing statements about peaceful acceptance of a poor prognosis. We identified a number of studies focusing on acceptance of a poor prognosis or death [14, 25, 34, 43, 44, 46, 50, 52]. We suggest a focused review of these studies in order to clarify the nature of peaceful acceptance of a poor prognosis or death in patients with cancer. The current review can serve as background knowledge in doing so.

Clinicians play an important role in the management of distress in patients with cancer [72, 73]. A better understanding of acceptance may assist clinicians in this task. The current review provides initial insights into how this might be accomplished, such as by encouraging patients to shift their focus or to relinquish control, or by fostering sharing with relatives and friends. However, a deeper understanding of acceptance, including its determinants and consequences, is needed to provide clear guidance for clinical practice.

In conclusion, acceptance of disease, cancer, or poor prognosis and imminent death is a relatively new area of research, characterized by significant heterogeneity in the definition and focus of acceptance, as well as in the hypothesized determinants and outcomes. To advance this field, it is essential to develop a generally accepted definition of acceptance and to consistently specify its focus. The present scoping review provides a solid foundation for this endeavor.

## Author Contributions

**Joost Dekker:** conceptualization, investigation, analysis, supervision, validation, writing – original draft preparation. **Chris Welling:** investigation, writing – review and editing. **Mariette Labots:** conceptualization, analysis, supervision, validation, writing – review and editing.

## Conflicts of Interest

The authors declare no conflicts of interest.

## Data Availability

The data that supports the findings of this study are available in Appendix B.

## Appendix A Search Strategy in PubMed

CANCER:

(“Neoplasms”[Mesh] Intitle OR neoplas*[Title] OR tumor*[Title] OR tumor*[Title] OR cancer*[Title] OR lymphoma*[Title] OR malignan*[Title] OR oncolog*[Title] OR carcinom*[Title] OR melanom*[Title]).

AND

ACCEPTANCE:

(Peace*[Title] OR peaceful[Title] OR acceptance[Title] OR calm[Title] OR serene*[Title] OR equanimity*[Title] OR harmony*[Title] OR tranquil*[Title] OR relaxed*[Title]).

## Appendix B Tables B1 and B2

**TABLE B1:** Information extracted from the studies.

No.	Authors	Year of publication	Title of article	Country	Aim of the study	Design	Sample size N	Type of cancer	Cancer‐stage	Time since diagnosis	Mean age (years)	% female
1	J. Hinton	1999	The progress of awareness and acceptance of dying assessed in cancer patients and their caring relatives.	UK	This enquiry assesses patients’ and relatives’ own accounts of how far they came to terms with death and investigates some potential influences.	Longitudinal	76	Lung (17), colorectal (11), stomach (11), breast (9) or other sites (28)	Palliative, terminal	NS	65 (range 33–84)	44.7
2	A. Czerw, U. Religioni, A. Deptata	2016	Assessment of pain, acceptance of illness, adjustment to life with cancer and coping strategies in breast cancer patients	Poland	The purpose of the study was to evaluate coping strategies, pain management, disease acceptance, and adjustment to cancer in patients diagnosed with breast cancer.	Cross‐sectional	193	Breast cancer	NS	NS	NS	100
3	A. Czerw, U. Religioni, K. Sygit, A. Nieradko‐Heluszko, D. Mękal, O. Partyka, M. Mikos, M. Eid, L. Strzępek, T. Banaś	2021	Pain Control, Acceptance and Adjustment to the Disease among Patients with Ovarian, Endometrial and Breast Cancer in Poland	Poland	The aim of the study was to assess the location of pain control, strategies of coping with pain, the level of acceptance of cancer, and adjustment to life with disease of women with ovarian, endometrial and breast cancer.	Cross‐sectional	481	Ovarian (35.6%), endometrial (24.1%) and breast cancer (40.1%)	Ended oncological treatment	NS	56.6 (range 21–96)	100
4	A. Czerw, U. Religioni, P. Szumilas, K. Sygit, O. Partyka, D. Mękal, S. Jopek, M. Mikos, L. Strzępek	2022	Normalization of the AIS (Acceptance of Illness Scale) questionnaire and the possibility of its use among cancer patients	Poland	The aim of the study is evaluation of the use of the Acceptance of Illness Scale (AIS) among people with cancer.	Cross‐sectional	1187	Women: breast 29%, ovarian 25.8%, endometrial 17.4%, colorectal 14.9%, stomach 6.8%, pancreatic 3.6% and bladder cancer 2.6%. Men: prostate 43.8%, colorectal 26.9%, bladder 15.9%, stomach 9.2%, pancreatic X	NS	NS	62 (range 21–96)	56.1
5	A. Nowicki, P. Graczyk, M. Lemanowicz	2017	The acceptance of illness in lung cancer patients before and after surgical treatment	Poland	To assess the acceptance of disease in patients before and after lung cancer surgery	Longitudinal observational	87	Lung cancer	Early staged, operated	NS	30–49: 10.3%, 50–69: 66.7%, > 70: 23.0%	24.1
6	A. Nowicki, P. Sianoszek, P. Farbricka	2019	Sense of coherence and acceptance of the disease in patients with lung cancer during palliative chemotherapy	Poland	The primary objective was to assess the level of sense of coherence and acceptance of the disease in patients with lung cancer during palliative chemotherapy as well as coherence and acceptance together with socio‐demographic factors.	Cross‐sectional	100	Lung cancer	Palliative	NS	62.8 (range 50–65)	34
7	A. Ray, S. D. Block, R. J. Friedlander, B. Zhang, P. K. Maciejewski, H. G. Prigerson	2006	Peaceful Awareness in Patients with Advanced Cancer	USA	Our hypothesis was that patients who were both peaceful and aware of their terminal prognosis would represent the optimal case in which both mental health and positive end‐of‐life care outcomes are retained.	Cross‐sectional (patients) and longitudinal (caregivers)	280	NA	Advanced cancer (presence of distant metastases and failure of first‐line chemotherapy).	NS	57.3 (range 20–65+)	45.6
8	A. Czerw, M. Bilinska, A. Deptata	2016	The assessment of the impact of socio‐economic factors in accepting cancer using the Acceptance of Illness Scale	Poland	Examining the level of acceptance of illness in cancer patients	Cross‐sectional	74	Pancreatic and colorectal cancer	Metastatic (32) and non‐metastatic (38)	0–1 years: *n* = 37, 2 years: *n* = 17, 2+ years: *n* = 17	< 55: 32.4%, 55–65: 38%, > 65: 29.6%	29 women, 42 men (71?)
9	A. Czerw, U. Religioni, A. Deptata, A. Fronczak	2017	Pain, acceptance of illness, adjustment to life with cancer and coping strategies in prostate cancer patients	Poland	The purpose of the study was to evaluate coping strategies, pain management, illness acceptance, and adjustment to cancer in patients diagnosed with prostate carcinoma and the effect of socioeconomic variables on the above‐mentioned issues.	Cross‐sectional	228	Prostate cancer	NS	NS	NS	0
10	B. Jankowska‐Polańska, N. Świątoniowska‐Lonc, E. Ośmiałowska, A. Gałka, M. Chabowski	2020	The association between Illness Acceptance and Quality of Life in Women with Breast Cancer	Poland	The aim of the study was to investigate the association between illness acceptance and quality of life (QoL) in patients with breast cancer.	Cross‐sectional	150	Breast cancer	Operated	0–2 years: 45%, 2–5 years: 34%, 5+ years: 31%	53.1 (range 26–77)	100
11	C. E. Mosher, E. Krueger, E. Secinti, S. A. Johns	2021	Symptom experiences in advanced cancer: relationships to acceptance and commitment therapy constructs	USA	This study examined relations between acceptance and commitment therapy (ACT) constructs and symptom‐based subgroups of advanced cancer patients.	Cross‐sectional	201	Breast, gastrointestinal, lung or prostate cancer	Advanced. At least 3 weeks post‐diagnosis of stage IV cancer or completed primary treatment for stage I or II cancer at least 6 months ago	NS	62	0.49
12	C. F. Pereira, K. Cheung, E. Alie, J. Wong, M. J. Esplen, Y. W. Leung	2021	Pathways to Acceptance in Participants of Advanced Cancer Online Support Groups	Canada	The aim of the article is to map ways that people reach acceptance with advanced cancer by analysing group discussions	Cross‐sectional, qualitative	18	Breast, colorectal, head and neck, other cancers	Advanced	NS	Majority 55–64 years (25–65+)	94.4
13	C. G. Jensen, P. Elsass, L. Neustrup, T. Bihal, H. Flyger, S. M. Kay, S. Khan, S. S. Jensen, A. Pedersen, H. Würtzen	2014	What to listen for in the consultation. Breast cancer patients’ own focus on talking about acceptance‐based psychological coping predicts decreased psychological distress and depression	Denmark	To analyze whether qualitative themes in breast cancer patients’ self‐presentations predicted symptoms of psychological distress and depression in order to improve the consultation process.	Cross‐sectional qualitative and cross‐sectional quantitative	97	Breast cancer	NS	Mean: 8.6 months	54.2 (range 28–72)	100
14	D. Branecka‐Wozniak, A. Cymbaluk‐Ploska	2021	The influence of support and medical data on the level of illness acceptance, the way of coping with a stressful situation, and mental adjustment to the disease among cancer patients	Poland	The aim of this study was to analyze the behavior of cancer patients.	Cross‐sectional	145	Tumors from the gastrointestinal (56; 30%), genitourinary (42; 23%), other (32; 17%) and respiratory system (14; 8%).	Malignant (89%), benign (11%)	48% more than 1 year; 27% for 1–2 years, 16% for 3–4 years, and 9% > 5 years.	NS	NS
15	M. Chabowski, J. Polański, B. Jankowska‐Polanska, K. Lomper, D. Janczak, J. Rosinczuk	2017	The acceptance of illness, the intensity of pain and the quality of life in patients with lung cancer	Poland	The aim of the study was to examine the relationship between pain, acceptance of illness and quality of life	Cross‐sectional	155	Lung cancer	Both metastatic and non‐metastatic	NS	62.23	48.4
16	Y. Y. Chen, M. Ahmad, F. B. Ismail	2019	Illness acceptance as mediator for cancer‐related complaints and psychological distresses among Malaysian cancer patients	Malaysia	Aimed to investigate the relationship between psychological problems, illness acceptance and cancer‐related complaints among Malaysian cancer patients	Cross‐sectional	106	Breast cancer (51%), colon (10%), prostate (5%), lung (3%)	NS	Mean: 4.90 years (SD = 4.39)	61.6	81
17	K. Chinh, E. Secinti, S. A. Johns, A. T. Hirsh, K. D. Miller, B. Schneider et al.	2020	Relations of Mindfulness and Illness Acceptance With Psychosocial Functioning in Patients With Metastatic Breast Cancer and Caregivers	USA	To examine relationships in mindfulness and illness acceptance and psychosocial functioning in patients with metastatic breast cancer and their family caregivers.	Cross‐sectional	33	Breast cancer	Metastatic (stage IV)	Mean 4.48 years (SD = 3.85)	55.27 (SD = 11.49)	100
18	E. Cipora, M. Konieczny, J. Sobieszczański	2018	Acceptance of illness by women with breast cancer	Poland	The objective of the study was determination of the level of acceptance of illness by women treated for breast cancer.	Cross‐sectional	231	Breast cancer	NS, receiving treatment	NS	56.23	100
19	T. N. Elumelu, C. C. Asuzu, E. O. Akin‐Odanye	2015	Impact of active coping, religion and acceptance on quality of life of patients with breast cancer in the department of radiotherapy, UCH, Ibadan	Nigeria	The study seeks to assess the impact of active coping, religion and acceptance on the QoL of patients with breast cancer.	Cross‐sectional	110	Breast cancer	1/2 (40,9%), 3/4 (59,1%)	NS	46.82 (SD = 10.55)	96.4
20	P. Jimenez‐Fonseca, U. Lorenzo‐Seva, P. J. Ferrando, A. Carmona‐Bayonas, C. Beato, T. García, M. D. M. Muñoz, A. Ramchandani, I. Ghanem, A. Rodríguez‐Capote et al.	2018	The mediating role of spirituality (meaning, peace, faith) between psychological distress and mental adjustment in cancer patients	Germany	The first aim of this study was to analyze the psychometric properties of the FACIT‐Sp Spanish version scale (dimensionality, structure, and reliability of the derived scores). The second aim was to establish the mediating effect of spiritual wellbeing on psychological distress and mental adjustment	Cross‐sectional	504	Localized, non‐advanced solid tumors treated with surgery; colon (40,5%), breast (33,7%), and other cancers	Non‐advanced, I–II (58,1%), III (41,9%)	NS	58.6 (SD = 12.1)	61.5
21	K. Suwannapong, S. Thanasilp, D. L. Doutrich, L. Akkayagorn, N. H. Long, J. Chimluang, N. Pudtong, R. Upasen	2022	Describing Death Acceptance Among Thai Buddhists With Cancer.	Thailand	The purposes of this study were to describe Death Acceptance among Thai Buddhists with cancer and to compare Death Acceptance differences in demographic data	Cross‐sectional	363	Breast 28.10%, Gastrointestinal/Liver 24.52%, Lung 13.77%, Urogenital/Reproductive 11.85%, Lymphoma/Acute Myeloid Leukemia 10.74%, Head‐and‐Neck/Neuro 11.85%, other 0.55%	I/II (32.8%), III (25.1%), IV 42.2%)	Less than 6 months: 39.9%, more than 6 months: 60.1%	53.73 (SD = 11.58)	63.1
22	W. Krajewski, M. Mazur, A. Poterek, A. Pastuszak, U. Halska, A. Tukiendorf, J. Rymaszewska, R. Zdrojowy R	2018	Assessment of Pain Management, Acceptance of Illness, and Adjustment to Life with Cancer in Patients with Nonmuscle Invasive Bladder Cancer	Poland	The purpose of the study was to evaluate pain management, disease acceptance, and adjustment to cancer in homogenous group of patients diagnosed with nonmuscle‐invasive bladder cancer (NMIBC). We also analysed the effect of socioeconomic and clinical variables on the abovementioned issues.	Cross‐sectional	252	Nonmuscle invasive bladder cancer	0: 14%, A: 44%, 1: 22%, 2: 12,1%, Other than UCC: 2,4%; missing: 1,6%, CIS: 3,2%.	NS	< 65: 37,7%, 65–70: 27%, 70–75: 14,3%, >75: 21%	0
23	D. Krok, E. Telka, M. Moroń	2023	Marital satisfaction, partner communication, and illness acceptance among couples coping with breast cancer: A dyadic approach	Poland	This study examines whether partner communication mediates relationships between marital satisfaction and illness acceptance among couples coping with breast cancer within a dyadic approach.	Cross‐sectional	136	Breast cancer	Stages I, II, and III	Mean 3.32 years (SD = 1.45)	51.5 (SD = 11.9)	100
24	D. Krok, E. Telka, B. Zarzycka	2022	Total Pain and Illness Acceptance in Pelvic Cancer Patients: Exploring Self‐Efficacy and Stress in a Moderated Mediation Model	Poland	The present prospective, longitudinal study examined the relationship between total pain and illness acceptance among pelvic cancer patients, taking into consideration the moderated mediation effects of self‐efficacy and stress.	Longitudinal observational	267	Pelvic cancer	Completed radiotherapy and positive prognosis	NS	61.26 (SD = 12.77)	100
25	K. Kuba, G. Weißflog, H. Götze, F. García‐Torres, A. Mehnert, P. Esser	2019	The relationship between acceptance, fatigue, and subjective cognitive impairment in hematologic cancer survivors.	Germany	The present study aims to investigate the correlation between acceptance and fatigue and subjective cognitive impairment in hematologic cancer survivors.	Cross‐sectional	922	Hematologic cancer	In remission (69%), second tumor (17%).	Mean: 8.9 years (SD = 4.5)	64 years (SD = 13.4)	43
26	J. W. Mack, M. Nilsson, T. Balboni, R. J. Friedlander, S. D. Block, E. Trice, H. G. Prigerson	2008	Peace, Equanimity, and Acceptance in the Cancer Experience (PEACE): validation of a scale to assess acceptance and struggle with terminal illness	USA	The authors developed a measure of peaceful acceptance at the EOL, and evaluated the role of peaceful acceptance in EOL decision‐making and care.	Cross‐sectional	160	NA	Advanced cancer (expected prognosis < 1 year) with failure of first‐line chemotherapy.	NS	63 (SD = 12.5)	50
27	S. L. Manne, D. A. Kashy, S. Virtue, K. R. Criswell, D. W. Kissane, M. Ozga, C. J. Heckman, J. Stapleton, L. Rodriguez	2018	Acceptance, social support, benefit‐finding, and depression in women with gynaecological cancer	USA	This study's goal was to use a multiple mediator model to evaluate whether the effect of benefit‐finding on depression was mediated by acceptance of cancer, acceptance of emotions, and received social support.	Cross‐sectional	174	Gynaecological cancers	I (14.4%), II (13.8%), III (49.4%), IV (21.3%)	Mean: 3.9 months (SD = 1.9)	55.32 (SD = 10.28)	100
28	T. V. Merluzzi, N. Salamanca‐Balen, E. J. Philip, J. M. Salsman	2023	"Letting go" – Relinquishing control of illness outcomes to God and quality of life: Meaning/peace as a mediating mechanism in religious coping with cancer	USA	Meaning/Peace, as well as coping efficacy were hypothesized to be mediators and, thus, explanatory mechanisms in the relationship between relinquishing control to God and quality of life outcomes.	Cross‐sectional	548	Breast (40.6%), lung (6.4%), prostate (7.2%) colorectal (7.4%), lymphoma (4.4%), female reproductive (8.0%), other (27.4%)	NS	NS	63.07 (range 24–89)	66.8
29	M. Pasek, G. Dębska, E. Wojtyna	2017	Perceived social support and the sense of coherence in patient‐caregiver dyad versus acceptance of illness in cancer patients	Poland	The aim of this study was to analyse direct and indirect interrelationships between perceived support and the sense of coherence in patient–caregiver dyad, and acceptance of illness in cancer patients.	Cross‐sectional	80	NA	NS	Mean 6.53 months (SD = 2.09)	59.05 (11.13)	57.5
30	R. Philipp, A. Mehnert, C. Lo, V. Müller, M. Reck, S. Vehling	2019	Characterizing death acceptance among patients with cancer	Germany	We aimed to broaden the understanding of death acceptance by exploring associated demographic, medical, and psychological characteristics.	Longitudinal observational	307	Breast (39%), lung (36%), gynaecological (14%), gastrointestinal (11%)	Early (49%), advanced (51%)	Mean 32.1 months (SD54.2)	59.6 (SD = 11.1)	69
31	M. Pinquart, C. Fröhlich, R. K. Silbereisen, U. Wedding	2005	Death acceptance in cancer patients	Germany	In the present study we ask a) whether the level of death acceptance would differ between cancer patients and healthy control group members and between younger and older participants; b) which illness‐specific, sociodemographic, and psycho‐logical variables relate to higher levels of cancer patients’ death acceptance; and c) whether death acceptance of cancer patients would change during the course of therapy.	Longitudinal observational	337	Hematological and other cancers	32.5% curative care, rest palliative care	Mean: 2 weeks	57.8 (SD = 14.9)	41
32	J. Polański, M. Chabowski, B. Jankowska‐Polańska, D. Janczak, J. Rosińczuk	2018	Histological subtype of lung cancer affects acceptance of illness, severity of pain, and quality of life	Poland	To evaluate differences in acceptance of illness, level of perceived pain, and quality of life (QoL) between patients with small‐cell lung cancer (SCLC) and nonsmall‐cell lung cancer (NSCLC).	Cross‐sectional	257	Lung cancer	No metastases (60.7%), metastases to one organ (26.1%), multiorgan (13.2%)	NS	between 62.8 and 64.1'	44.8
33	R. M. Quinto, F. De Vincenzo, L. Campitiello, M. Innamorati, E. Secinti, L. Iani	2022	Meaning in Life and the Acceptance of Cancer: A Systematic Review	Italy	This study provides a systematic review of the associations between meaning in life and the acceptance of cancer in cancer patients.	Systematic review	464 from 3 articles	Breast and/or cervical cancer	NS	Mostly under 2 years	Mean between 53.4 and 54 years.	100
34	R. Upasen, S. Thanasilp, L. Akkayagorn, J. Chimluang, W. Tantitrakul, D. L. Doutrich, W. Saengpanya	2022	Death Acceptance Process in Thai Buddhist Patients With Life‐Limiting Cancer: A Grounded Theory	Thailand	This study explores the death acceptance process among patients with life‐limiting cancer.	Cross‐sectional, Qualitative	13	NA	III or IV, palliative	NS	NS	69.2
35	U. Religioni, A. Czerw, A. M. Badowska‐Kozakiewicz, A. Deptała	2020	Assessment of Pain, Acceptance of Illness, Adjustment to Life, and Strategies of Coping with Illness among Patients with Gastric Cancer	Poland	The objective of the study was to evaluate the strategy of coping with pain and its control, acceptance of illness, and adjustment to living with cancer in patients suffering from gastric cancer.	Cross‐sectional	93	Gastric cancer	NS	NS	Mean: 59.98 (14.25) years	48.4
36	U. Religioni, A. Czerw, A. Deptała	2015	Acceptance of Cancer in Patients Diagnosed with Lung, Breast, Colorectal and Prostate Carcinoma	Poland	The aim of the study was to verify the influence of socioeconomic factors on acceptance of illness in patients suffering from breast, lung, colorectal and prostate carcinoma.	Cross‐sectional	902	Lung, breast, colorectal and prostate cancer	NS	NS	NS	NS
37	U. Religioni, A. Czerw, A. Deptala	2021	Assessment of Pain, Acceptance of Illness, Adaptation to Life, and Strategies of Coping With the Disease, in Patients With Bladder Cancer	Poland	To identify the socio demographic features linked acceptance and coping with the disease in patients suffering from bladder cancer.	Cross‐sectional	100	Bladder cancer	NS	NS	NS	NS
38	E. Sachs, E. Kolva, H. Pessin, B. Rosenfeld, W. Breitbart	2013	On sinking and swimming: the dialectic of hope, hopelessness, and acceptance in terminal cancer	USA	The aim of this study was to examine the relationship between hope and hopelessness in advanced cancer and to identify factors that maintain hope and increase vulnerability to hopelessness.	Cross‐sectional, qualitative	22	Lung, pancreatic, ovarian, prostate, breast cancer	IV	NS	58.5 (30–80) year	68.2
39	E. Secinti, D. B. Tometich, S. A. Johns, C. E. Mosher	2019	The relationship between acceptance of cancer and distress: A meta‐analytic review	USA	We first propose an integrated model of acceptance of cancer. Then we examine the strength of the relationships between acceptance of cancer and general and cancer specific distress in cancer patients and potential moderators of these relationships.	Meta‐analysis	15,488 from 78 articles	Mixed or breast cancer	25.9% advanced‐stage (stage III, IV/metastatic)	Mean: 2.16 years (SD = 1.78)	55.75 (SD = 8.52)	Mean 71,13%
40	E. Secinti, W. Wu, E. F. Krueger, A. T. Hirsh, A. M. Torke, N. H. Hanna, N. Adra, G. A. Durm, L. Einhorn, R. Pili et al	2022	Relations of perceived injustice to psycho‐spiritual outcomes in advanced lung and prostate cancer: Examining the role of acceptance and meaning making	USA	This study examined relations between cancer‐related perceived injustice and psycho‐spiritual outcomes as well as potential mediators of these relationships.	Cross‐sectional	201	Lung and prostate	Advanced	Mean: 3 years (SD = 2.7) since advanced cancer diagnosis	67 (SD = 11)	28
41	S. T. Tang, W. C. Chang, J. S. Chen, W. C. Chou, C. H. Hsieh, C. H. Chen	2016	Associations of prognostic awareness/acceptance with psychological distress, existential suffering, and quality of life in terminally ill cancer patients' last year of life	Taiwan	This study longitudinally evaluated the associations of accurate prognostic awareness and prognostic acceptance with psychological distress, existential suffering, and QOL while comprehensively controlling for confounders in Taiwanese terminally ill cancer patients’ last year of life.	Longitudinal observational	325	Stomach, liver, pancreas, heas and neck, lung	Terminal	Mean: 18.97 months (SD = 34.16)	69.2% < 66 years	42.5
42	G. N. Thompson, H. M. Chochinov, K. G. Wilson, C. J. McPherson, S. Chary, F. M. O'Shea, D. R. Kuhl, R. L. Fainsinger, P. R. Gagnon, K. A. Macmillan	2009	Prognostic acceptance and the well‐being of patients receiving palliative care for cancer	Canada	To identify the impact of prognostic acceptance/nonacceptance on the physical, psychological, and existential well‐being of patients with advanced cancer	Cross‐sectional	381	Lung, GI, breast, genitourinary, gynaecologic, other	Advanced, terminal (estimated prognosis of 6 months or less)	NS	between 59 and 68 years'	Between 55% and 61%'
43	H. Wang, X. Sun, H. Yue, Y. Yang, D. Feng	2022	The dyadic effects of personality traits on depression in advanced lung cancer patients and caregivers: The mediating role of acceptance of illness	China	This study aimed to explore the intrapersonal and interpersonal effects of three personality traits—neuroticism, extraversion and conscientiousness—on depression and examine whether acceptance of illness mediates the actor and partner effects in advanced lung cancer patients and caregivers using the Actor–Partner Interdependence Mediation Model (APIMeM).	Cross‐sectional	304	Lung cancer	III (32.6%) or IV (67.4%)	< 3 months: 45.1%, 3–6 months: 14.8%, 6–12 months: 16.1%, > 1 year: 24.0%	58.5 (SD = 10.1)	33.2
44	S. Wieder‐Huszla, J. Owsianowska, A. Chudecka‐Głaz, D. Branecka‐Woźniak, A. Jurczak	2022	The Significance of Adaptation and Coping with Disease among Patients with Diagnosed Gynaecological Cancer in the Context of Disease Acceptance	Poland	The aim of the study was to assess the influence of adaptation and coping with gynaecological cancer on the level of disease acceptance among the studied women.	Cross‐sectional	81	Uterine cancer, ovarian cancer	NS	NS	60.31 (11.72)	100
45	R. Piotrkowska, A. Kruk, A. Krzemińska, W. Mędrzycka‐Dąbrowska, K. Kwiecień‐Jaguś	2023	Factors Determining the Level of Acceptance of Illness and Satisfaction with Life in Patients with Cancer	Poland	The purpose of this work is to assess the acceptance of illness and satisfaction with life in patients with cancer, as well as to identify social, demographical, and clinical factors that significantly differentiate their acceptance of illness and satisfaction with life.	Cross‐sectional	120	Breast (16.7%), lung (24.2%), reproductive organs (15.0%), colectoral (10.8%), bone and multiple myeloma (2.5%), gastrointestinal (6.7%), skin (8.3%), urinary (6.7%), other (8.5%)	NS	< 6 months: 45%, 6 months ‐ 1 year: 39.16%, over 3 years: 15.83%	56 (18–88)	55.8
46	S. Q. Chen, J. E. Liu, Z. Li, Y. L. Su	2017	The process of accepting breast cancer among Chinese women: A grounded theory study	China	To describe the process by which Chinese women accept living with breast cancer	Cross‐sectional, qualitative	18	Breast cancer	NS	NS	NS	100
47	S. Fawson, Z. Moon, K. Novogrudsky, F. Moxham, K. Forster, I. Tribe, R. Moss‐Morris, C. Johnson, L. D. Hughes	2024	Acceptance and commitment therapy processes and their association with distress in cancer: a systematic review and meta‐analysis	UK	To identify the strength of associations between ACT processes (including the ACT adjunct process of self‐compassion) and distress (e.g., depression, anxiety, emotional wellbeing) in patients with cancer using meta‐analyses where possible and narrative review where there are insufficient studies for meta‐analysis. The secondary objective is to: identify which ACT processes mediate distress outcomes in ACT interventions for patients with cancer.	Systematic review and meta‐analysis	17,195 participants (108 samples). Studies on acceptance: sample sizes ranged from 14 to 460.	Studies on acceptance: breast cancer (*n* = 14), mixed cancer samples (*n* = 10), gynaecological (*n* = 3), and one study each in head and neck, brain, gastrointestinal, blood, colorectal and melanoma.	Mixed stages	Overall: ranging from 25 days to 10 years.	Ranging from 37.9 to 72.18	Ranging from 0% to 100%
48	D. Krok, E. Telka, D. Kocur	2024	Perceived and Received Social Support and Illness Acceptance Among Breast Cancer Patients: The Serial Mediation of Meaning‐Making and Fear of Recurrence	Poland	To investigate whether meaning‐making and fear of recurrence serially mediated the relationship between perceived and received social support and illness acceptance.	Cross‐sectional	246	Breast cancer	NS	Illness duration: M = 3.59, SD = 4.51 (years?)	NS	100
49	D. Krok. E. Telka, M. Moron	2024	Dyadic Effects of Attachment on Illness Acceptance in Patients with Breast Cancer and Spousal Caregivers: Sense of Coherence as a Mediator	Poland	We aimed to examine whether sense of coherence mediates the association of different attachment styles with illness acceptance experienced in breast cancer dyads (women with breast cancer and their spouses).	Cross‐sectional	145 patients and 145 partners	Breast cancer	Stage I–III	Diagnosed within 6 months	51.6 (SD = 10.59) years	100
50	A. Królikowska, M. Stanisławska, M. Starczewska, A. Rybicka, K. Rachubinska	2024	In Search of Variables Affecting Mental Adjustment and Acceptance of Cancer among Urological Patients	Poland	The aim of the present study was the search for variables affecting the acceptance and mental adjustment to cancer in patients undergoing surgical urological treatment.	Cross‐sectional	150	Genitourinary cancer	NS	NS	62	29.3
51	M. Matbouei, M. Samsami, M. Soleimani	2024	Breast Cancer Survivors’ Experiences of Acceptance Following Recurrence	Iran	The aim of this study was to explore how patients experience breast cancer recurrence and go through a process of negotiating acceptance	Cross‐sectional, qualitative	16	Breast cancer	Recurrent disease	Recurrence detection time after the initial treatment ranging from 2 months to 20 years.	Ranging from 33 to 63 years.	100
52	C. Sauer, T. Hansen, H. G. Prigerson, J. W. Mack, T. J. Bugaj, G. Weißflog	2024	Peace, equanimity and acceptance in the cancer experience: validation of the German version (PEACE‐G) and associations with mental health, health‐related quality of life and psychological constructs	Germany	The aim of this study was to examine the psychometric properties of the German translation of the Peace, Equanimity, and Acceptance in the Cancer Experience (PEACE) questionnaire, and to investigate its associations with mental health, health‐related quality of life (HRQoL) and related constructs.	Cross‐sectional	213	Mixed	NS	3.09 years (SD = 4.10 years).	54.24 (SD = 11.50)	64
53	F. Uslu‑Sahan, N. Gulcan	2024	Supportive care needs in predicting the acceptance of illness among gynaecological cancer patients undergoing therapy: a cross‑sectional study	Turkey	To examine the effect of supportive care needs and related factors in the acceptance of illness	Cross‐sectional	190	Gynaecological cancer	Early (stages I and II): 63, Advanced (stages III and IV): 127	Onset of diagnosis (months) ≤ 12: 83, > 12: 107	59.01	100
54	B. Xu, M. Qian, L. Zhu, Z. Lu	2024	Study on the Relationship Between Uncertainty Tolerance and Positive Acceptance in Postoperative Patients With Cervical Cancer	China	To clarify the correlation between uncertainty tolerance and positive acceptance	Cross‐sectional	233	Cervical cancer	Mixed? b1 43 Ib2 91 IIa1 75 IIa2 24	NS	30: 17, 30–50: 156, 50: 60	100

No.	Authors	Year of publication	Definition of acceptance	Focus of acceptance	Measurement of acceptance	Acceptance at a specific point in care	Data on acceptance, reported as G = group average; N = number of accepting patients	Factors influencing acceptance	Association with metastatic disease	Association of acceptance with quality of life and functioning	Acceptance as a mediator
1	J. Hinton	1999	Acceptance was described in terms of death's inevitability, faith and spiritual values, life's diminishing rewards (lack of pleasure), completing life, final benefits, humour, sharing, final activities etc. Individuals often used more than one concept. It is explained as coming to terms with this prospect – or the opposite.	Death	Patients and relatives were interviewed separately each week for eight weeks and fortnightly from then on. Acceptance rating 1‐9 by researcher.	In the final weeks patients' lives.	N Stable level of acceptance 37 (51%), Growing acceptance 22 (31%), Fluctuating acceptance 7 (10%), Falling acceptance 6 (8%)	Patients level of acceptance was positively correlated with those of the partners. Patients were more accepting if female and if quality of life was high.	Only palliative/terminal patients	NS	NS
2	A. Czerw, U. Religioni, A. Deptata	2016	NS	Illness	Acceptance of Illness Scale (AIS)	NS	G 28.45 (7.98)	The higher the income the more acceptance of the disease. Patients living in large cities had higher scores and hence presented better adjustment to disease	NS	NS	NS
3	A. Czerw, U. Religioni, K. Sygit, A. Nieradko‐Heluszko, D. Mękal, O. Partyka, M. Mikos, M. Eid, L. Strzępek, T. Banaś	2021	NS	Illness	Approval of Illness Scale (AIS)	NS	G 27.48 (Mean AIS)	The highest average disease acceptance score characterizes patients with breast cancer. Higher education of patients, living in larger cities, and higher income per person in the household positively affected the acceptance of the disease by the patients. Patients with metastases and being subjected to chemotherapy scored lower.	People with metastases (M=25.19) scored lower on acceptance than patients without (M = 28.43).	NS	NS
4	A. Czerw, U. Religioni, P. Szumilas, K. Sygit, O. Partyka, D. Mękal, S. Jopek, M. Mikos, L. Strzępek	2022	Acceptance of illness is an emotional determinant of functioning with and adaptation to the illness and is manifested in a small intensity of negative emotions associated with the illness.	Illness	Acceptance of Illness Scale (AIS)	NS	G Women: 27.15, Men: 28.36	Acceptance was higher for: men, people with higher education, people living in big cities, with higher incomes, without metastases and not undergoing chemotherapy.	People with metastases (M = 25.21) scored lower on acceptance than patients without (M = 28.77).	NS	NS
5	A. Nowicki, P. Graczyk, M. Lemanowicz	2017	NS	Illness	Acceptance of Illness Scale (AIS)	Before and after surgery	G Before surgery: 26.2 (6.49), after surgery: 20.89 (6.44)	Acceptance decreases after surgery. Male patients, patients aged 50–69 years, with primary, middle or vocational education, employed persons show a significantly worse illness acceptance.	NS	NS	NS
6	A. Nowicki, P. Sianoszek, P. Farbricka	2019	Acceptance allows for a rational assessment of a difficult situation in which a patient is currently ill, which makes it possible to have a real impact on his/her health by taking up the fight against the disease.	Illness	Acceptance of Illness Scale (AIS)	NS	G and N 45% with the average total of 27.21 points.	We found no statistically significant correlation between the level of acceptance of the disease and sex, age, education, or place of residence.	NS	NS	NS
7	A. Ray, S. D. Block, R. J. Friedlander, B. Zhang, P. K. Maciejewski, H. G. Prigerson	2006	Acceptance is finding a deep sense of peace or harmony	Not specified	NIA/Fetzer Multidimensional Measure of Religiousness/Spirituality	NS	N Peaceful and Aware (17.5%), Peaceful Unaware (51.4%), Not peaceful and Aware (9.3%), Not peaceful ans Unaware (21.8%).	Caregivers of patients who were peacefully aware were significantly more likely to recall postloss that there was always a doctor in charge of the patient's care. They were also more likely to recall that a doctor or that medical staff spoke to the patient regarding treatment preferences.	Only palliative patients	Peaceful patients were more likely to feel “supported” and have a “wish to live”. Peacefully aware patients had lower rates of psychological distress, higher rates of advance care planning, and higher quality of death.	NS
8	A. Czerw, M. Bilinska, A. Deptata	2016	The assessment of acceptance of illness is conducted at two levels: emotional and cognitive‐behavioural.	Illness	Acceptance of Illness Scale (AIS)	NS	G 27.56 (8.52)	Acceptance was higher for non‐ metastasized patients. There were no effects found for age, gender, education and professional status.	People with metastases (M = 24.25) scored lower on acceptance than patients without (M = 30.71).	NS	NS
9	A. Czerw, U. Religioni, A. Deptata, A. Fronczak	2017	NS	Illness	Acceptance of Illness Scale (AIS)	NS	G 30.39	Higher acceptance with higher income and higher education.	NS	NS	NS
10	B. Jankowska‐Polańska, N. Świątoniowska‐Lonc, E. Ośmiałowska, A. Gałka, M. Chabowski	2020	NS	Illness	Acceptance of Illness Scale (AIS)	NS	NS	The level of illness acceptance varies depending on the treatment method, and is the lowest in the group of women having undergone a mastectomy, and the highest in patients after a mastectomy with immediate breast reconstruction.	NS	Acceptance of illness improves the QoL of women treated for breast cancer, regardless of the specific treatment method.	NS
11	C. E.Mosher, E. Krueger, E. Secinti, S. A. Johns	2021	Acceptance is full awareness of experiences without defense, and defusion involves noticing thoughts without getting caught up in their content.	Cancer, not specified	Peaceful Acceptance subscale of the Peace, Equanimity, and Acceptance in the Cancer Experience (PEACE)	NS	G Normal levels of symptoms: 17.74 (2.53), moderate level of symptoms 16.64 (3.88), high level of symptoms 15.12 (3.34)	Mild to moderate symptom levels are related to less acceptance of cancer and internal experiences (e.g., symptoms, thoughts, feelings).	Only patients with advanced cancer	NS	NS
12	C. F. Pereira, K. Cheung, E. Alie, J. Wong, M. J. Esplen, Y. W. Leung	2021	In the context of a terminal illness, acceptance is considered to be an active approach in which a person does not attempt to judge or change the course of their illness or their internal experience	Illness, cancer, death	Directed content analysis of transcripts	NS	NS	Four themes related to illness acceptance: (1) Facilitator‐Initiated Discussion, including sub‐themes of Mindfulness, Relaxation and Imagery, Changing Ways of Thinking, and Spirituality; (2) Personal attitudes, including sub‐themes of Optimism and Letting Go of Control; (3) Supportive Environment, including the sub‐themes of Providing Support to Others and Receiving Support from Others; and (4) Existential Experience, which included sub‐themes of Living with the Diagnosis for an Extended Amount of Time, Legacy and Death Preparations, and Appreciating life.	Only palliative patients	NS	NS
13	C. G. Jensen, P. Elsass, L. Neustrup, T. Bihal, H. Flyger, S. M. Kay, S. Khan, S. S. Jensen, A. Pedersen, H. Würtzen	2014	NS	Unspecified	Interview. Patients’, unstructured presentations of ‘themselves and their illness’. Acknowledging or accepting the situation as it is.	NS	N 59% mentioned acceptance	NS	NS	Patients’ focus on acceptance‐based coping significantly predicted decreased psychological distress and depression, respectively.	NS
14	D. Branecka‐Wozniak, A. Cymbaluk‐Ploska	2021	NS	Illness	Acceptance of Illnees Scale (AIS)	NS	G Mean 24.35 (AIS)	No statiscally significant relatiosnships reported.	Only patients with advanced cancer	NS	NS
15	M. Chabowski, J. Polański, B. Jankowska‐Polanska, K. Lomper, D. Janczak, J. Rosinczuk	2017	NS	Illness	Acceptance of Illness Scale (AIS)	NS	G Mean 27.1 (AIS)	NS	NS	The acceptance of illness significantly correlated with all the domains of quality of life.	NS
16	Y. Y. Chen, M. Ahmad, F. B. Ismail	2019	NS	Cancer, not specified	Peace, Equanimity, and Acceptance in the Cancer Experience measure (PEACE)	NS	G 16.41 (SD: 4,11) (Mean PEACE)	Type of cancer (Rectal best, ovarian cancer, breast cancer, prostate cancer and lastly lung cancer), Time since diagnosis (the more time after diagnosis the more acceptance), Cancer‐related complaints (the more complaints, the less acceptance).	NS	NS	lllness acceptance was shown to be a mediator of cancer‐related complaints and psychological distress.
17	K. Chinh, E. Secinti, S. A. Johns, A. T. Hirsh, K. D. Miller, B. Schneider et al.	2020	Illness acceptance is defined as a sense of peace in confronting mortality and the losses associated with the illness. This concept suggests that accepting the reality of a chronic or terminal illness can lead to better psychological outcomes for patients by fostering a more peaceful and less resistant mindset towards their condition.	Cancer, not specified	Peace, Equanimity, and Acceptance in the Cancer Experience measure (PEACE)	NS	G 16.33 (SD = 3.08)	NS	Only patients with metastatic cancer	Illness acceptance, along with mindfulness, was found to be associated with reduced anxiety and depressive symptoms for both patients and caregivers.	NS
18	E. Cipora, M. Konieczny, J. Sobieszczański	2018	Acceptance of illness is coming to terms with own state of health, it enables the actual assessment of one's own health situation, and often provides incentives to struggle against the disease.	Illness	Acceptance of Illness Scale (AIS)	NS	G 26.53 (SD = 7.71)	The level of acceptance of cancer depended on the respondents’ age and their occupational situation. Younger women obtained a higher level of acceptance of their illness, compared to those who were older. It was confirmed that occupational activity was an important factor in obtaining higher values according to the AIS Scale by the women in the study. Such demographic characteristics as the place of residence, marital status, education level, and type of the occupation performed had no significant effect on the level of acceptance of the cancer.	NS	NS	NS
19	T. N. Elumelu, C. C. Asuzu, E. O. Akin‐Odanye	2015	Acceptance of a chronic health condition is a coping strategy that includes both direct action and passive components. Acceptance is defined as acknowledging the reality of the illness and learning to live with it, rather than resigning to fate. It involves recognizing that medical or behavioral therapies may not eradicate the health condition entirely and shifting focus from pain and other symptoms to other aspects of life.	Not specified	Brief Cope questionnaire	NS	G and N 63/110 = 57% used acceptance coping	NS	NS	Acceptance coping was associated with better social/family wellbeing, functional well‐being, overall quality of life.	NS
20	P. Jimenez‐Fonseca, U. Lorenzo‐Seva, P. J. Ferrando, A. Carmona‐Bayonas, C. Beato, T. García, M. D. M. Muñoz, A. Ramchandani, I. Ghanem, A. Rodríguez‐Capote et al.	2018	NS	Illness, not specified	Factor labeled meaning/peace in the Functional Assessment of Chronic Illness‐Spiritual WellBeing (FACIT‐Sp) scale.	After surgery	NS	NS	Only patients with not metastatic disease	NS	NS (mediation only reported for the total spirituality scale)
21	K. Suwannapong, S. Thanasilp, D. L. Doutrich, L. Akkayagorn, N. H. Long, J. Chimluang, N. Pudtong, R. Upasen	2022	Thai Buddhism encourages people to think of their death as a natural process and be aware that they must die someday; to understand it is impossible for them to go beyond death.	Death	Buddhist Death Acceptance Scale	NS	G Participants’ overall DA was at a high level (M = 3.34, SD = 0.43) according to BDAS. When considering its dimensions, the results showed that acceptance of the “natural process of death” dimension was highest scoring dimension (M = 3.37, SD = 0.52), followed by “preparing for death” (M = 3.27, SD = 0.61).	Patients age and cancer stage affected their DA, older patients had higher levels of acceptance, and patients with non‐advanced stage cancer had higher levels of acceptance.	Patients with non‐metastatic cancer scored higher on death acceptance	NS	NS
22	W. Krajewski, M. Mazur, A. Poterek, A. Pastuszak, U. Halska, A. Tukiendorf, J. Rymaszewska, R. Zdrojowy R	2018	NS	Illness	Acceptance of Illness Scale (AIS)	Patients scheduled for a transurethral resection of bladder tumour	G 28.8 (SD = 7.54)	AIS result was influenced by patient's marital status, yet, not by education, place of residence nor any clinical factor.	Only patients with non‐metastatic disease	AIS is correlated with a better and more constructive way of coping with the disease. AIS was also positively associated with pain control and pain reduction scores.	X
23	D. Krok, E. Telka, M. Moroń	2023	Coming to terms with one's own state of health	Disease	The Acceptance of Life with the Disease	NS	G 3.71 (SD = 0.69)	Patients with higher marital satisfaction had better supportive self‐communication, which in turn was associated with their higher illness acceptance. Patients with higher marital satisfaction had better own and spouse's supportive communication, which in turn was related to higher patient illness acceptance.	NS	NS	NS
24	D. Krok, E. Telka, B. Zarzycka	2022	One's adaptation to the disease in terms of the ability to accept health conditions and maintain overall life satisfaction.	Disease	The Acceptance of Life with the Disease	After radiotherapy	G 2.88 (SD = 0.50)	The more physical, psychological, social, and spiritual pain symptoms the patients experienced, the less they accepted negative health conditions and the effects of their illness. Stress moderated the indirect effect between total pain dimensions and illness acceptance through selfefficacy, but it did not moderate the relationship between total pain and illness acceptance	Only non‐metastatic patients	NS	NS
25	K. Kuba, G. Weißflog, H. Götze, F. García‐Torres, A. Mehnert, P. Esser	2019	Acceptance is psychological flexibility. Acceptance is defined by staying in contact with the present moment, which means to accept any emotional, cognitive or physical experiences without regulating or controlling them. Furthermore, it entails commitment, which means that behavior persists or is changed in order to pursue personal values and goals.	Not specified	Acceptance and Action Questionnaire (AAQ‐II)	After successful treatment, cancer survivors.	G Mean 4.80 (SD = 1.17) (Scale 1–7, high score means high acceptance)	NS	Only patients with non‐metastatic cancer (cancer survivors)	Survivors who habitually have higher levels of acceptance show fewer symptoms of fatigue and subjective cognitive impairment. The relationship between fatigue severity and impairment is significantly weaker when acceptance is high.	NS
26	J. W. Mack, M. Nilsson, T. Balboni, R. J. Friedlander, S. D. Block, E. Trice, H. G. Prigerson	2008	The patient's sense of acceptance, calmness, and peace. The emotional experience of coming to accept one's terminal illness.	Cancer, not specified	Peace, Equanimity, and Acceptance in the Cancer Experience (PEACE)	Prognosis of < 1 year	NS	Older patients scored higher on peaceful acceptance. Patients with less psychiatric ilness, more spirituality and less symptom burden had greater peaceful acceptance. Patients with feeding tubes had lower Peaceful Acceptance scores.	Only patients with metastatic cancer.		NS
27	S. L. Manne, D. A. Kashy, S. Virtue, K. R. Criswell, D. W. Kissane, M. Ozga, C. J. Heckman, J. Stapleton, L. Rodriguez	2018	Acceptance consists of accepting that the event has occurred as well as emotional acceptance, which is a willingness to feel one's positive and negative emotions without trying to control, change, or reject these feelings.	Not specified	Acceptance subscale of the COPE.	NS	NS	NS	No difference found in acceptance between metastatic and non‐metastatic disease	NS	Acceptance of emotion accounted for about half of the total indirect effect of benefit finding on depression, and the other two mediators (acceptance coping and social support) each account for approximately a fourth of that total indirect effect.
28	T. V. Merluzzi, N. Salamanca‐Balen, E. J. Philip, J. M. Salsman	2023	Religious and spiritual processes	Illness, not specified	Meaning and Peace subscales of the FACIT‐sp	NS	G 31.03 (SD = 5.79)	NS	NS	NS	As hypothesized, with all outcomes, Relinquishing Control was related to Meaning/Peace, which was related to Coping Efficacy, and, in turn, to outcomes. Finally, as hypothesized, in a reduced model, Meaning/Peace mediated the relationship between Relinquishing Control and all three outcomes
29	M. Pasek, G. Dębska, E. Wojtyna	2017	Acceptance of illness, which can be defined as approval of the fact of being ill, realising the need to adapt to the condition, tolerance to its unexpected and uncontrolled manifestations, and a decision to cope with unfavourable consequences of experienced ailments.	Illness	Acceptance of Illness Scale (AIS)	NS	G 26.63 (SD = 6.04)	Acceptance of illness in cancer patients depends on the sense of coherence and perceived social support, both their own and their caregivers.	NS	NS	NS
30	R. Philipp, A. Mehnert, C. Lo, V. Müller, M. Reck, S. Vehling	2019	Death acceptance refers to the absence of fear and anxiety about death and the acceptance of death as a natural aspect of life.	Death	Death acceptance subscale of the Life Attitude Profile‐Revised (LAP‐R)	NS	G 4.33 (SD = 1.3)	At baseline, mean death acceptance was 4.33 (standard deviation [SD] = 1.3, range 1–7). There was no change to follow‐up (P = 0.26). When all variables were entered simultaneously, patients who experienced high death acceptance were more likely to be older, male, widowed, and diagnosed with stage IV. They were less likely to be diagnosed with lung cancer and their death acceptance was lower with every month since diagnosis.	More acceptance in people with metastatic or advanced cancer	High death acceptance predicted lower demoralization and anxiety at follow‐up but not depression.	NS
31	M. Pinquart, C. Fröhlich, R. K. Silbereisen, U. Wedding	2005	In the present study, death acceptance is defined as a calm attitude toward the finiteness of one's life, and seeing death as an unchangeable fact of life that also might have some positive aspects	Death	Five item likertscale by Brandstadter	At the start of chemotherapy	G M = 14.9 (SD = 5.6)	Higher age but not having cancer was associated with higher levels of death acceptance, and differences in death acceptance between patients and healthy control group members were stronger in younger than in older participants. Within the patient sample, stronger physical impairments, male gender, having no long‐term goals, and religiosity were related to higher levels of death acceptance. Across a nine‐month interval, no changes in the mean level of death acceptance appeared. Nonetheless, younger patients with stronger physical impairments at T₁ showed a decline in death acceptance at T₂. We conclude that reasons that make illness and mortality salient reduce death acceptance in younger patients, but not in older patients.	Poorer prognosis did not make recently diagnosed patients more accepting to death		NS
32	J. Polański, M. Chabowski, B. Jankowska‐Polańska, D. Janczak, J. Rosińczuk	2018	NS	Illness	Acceptance of Illness Scale (AIS)	NS	G SCLC: 24.58 (8.73), NSCLC: 27.05 (9.06)	People with SCLC show lower levels of acceptance than people with NSCLC. Severity of many symptoms was significantly correlated with AIS score.	NS	There was a significant correlation between AIS score and all functioning scores measured with QLQ‐C30 in both study groups.	NS
33	R. M. Quinto, F. De Vincenzo, L. Campitiello, M. Innamorati, E. Secinti, L. Iani	2022	An active willingness to be present with cancer‐related realities while giving up efforts to judge or control cancer‐related appraisals or feelings.	Not specified, illness	Acceptance subscale of the Brief COPE (B‐COPE), Acceptance of Disability Scale‐Revised, Acceptance of Illness Scale (AIS)	NS	NS	People with higher sense of meaning in life had higher acceptance of their illness.	NS	NS	NS
34	R. Upasen, S. Thanasilp, L. Akkayagorn, J. Chimluang, W. Tantitrakul, D.L. Doutrich, W. Saengpanya	2022	Death acceptance is a dynamic and iterative process that involves three phases, namely, engaging suffering, being open‐minded about death, and adhering to Buddhist practices for increasing death consciousness.	Death	Interview	NS	Thai Buddhist patients with cancer in palliative care process death acceptance through three dynamic phases: engaging suffering, being open‐minded about death, and adhering to Buddhist practices for increasing death consciousness.	NS	Only patients with metastatic cancer.	NS	NS
35	U. Religioni, A. Czerw, A. M. Badowska‐Kozakiewicz, A. Deptała	2020	NS	Illness	Approval Illness Scale (AIS)	NS	G M = 24.02 (SD = 7.69)	None of variables examined affected the level of acceptance (age, place of living, education, treatment, work). Chemotherapy in the last year also did not affect acceptance of illness among the patients.	NS	NS	NS
36	U. Religioni, A. Czerw, A. Deptała	2015	Acceptance of disease is simultaneously conducted at two levels: the emotional and cognitive‐behavioral one. Patients learn to accept not only the symptoms but also the resulting changes in the quality of life, limitation of self‐reliance and independence, and thus the change of their individual roles in their families and the society.	Illness	Acceptance of illness scale (AIS)	NS	G M = 27.33 (SD = 8.44)	The level of acceptance of illness depends on the primary site of cancer. Prostate carcinoma patients show the highest and lung carcinoma patients the poorest acceptance of illness. The degree of disease acceptance depends on respondent's income. The higher the net income‐per‐household‐member, the better disease acceptance. Chemotherapy administration positively affects acceptance of illness amongst cancer patients.	NS	NS	NS
37	U. Religioni, A. Czerw, A. Deptala	2021	NS	Illness	Acceptance of illness scale (AIS)	NS	G M = 27.25 (SD = 7.52)	Income: higher income means higher acceptance. No influence found for: age, gender, education, place of recidence, work, chemotherapy or not.	NS	NS	NS
38	E. Sachs, E. Kolva, H. Pessin, B. Rosenfeld, W. Breitbart	2013	Acceptance implies a realistic understanding of prognosis and a willing relinquishing of inflexible or unrealistic hopes.	Death, poor prognosis	Semi‐structured interview	Life expectancy up to 12 months	Acceptance is the opposite of hopelessness. (is dit niet onderdeel van de definitie?, mlb)	These patients described that the introduction of adequate palliative care dissipated hopelessness, and in some cases resulted in new feelings of acceptance and hope for realistic goals.	Only metastatic cancer.	Acceptance of one's terminal prognosis was related to more adaptive forms of hope, such as hope for connection with others, enjoyment of daily pleasures, and investment in legacy projects, rather than hope for extended life.	X
39	E. Secinti, D. B. Tometich, S. A. Johns, C. E. Mosher	2019	Broadly, we conceptualize acceptance of cancer as an active willingness to be present with cancer‐related realities while giving up efforts to judge or control cancer‐related appraisals or feelings. In addition, acceptance of cancer involves a behavioral willingness in response to cancer‐related stressors, resulting in action aligned with deeply held values.	Not specified, illness, cancer	BCOPE‐A = Brief COPE ‐ Acceptance Subscale; COPE‐A = COPE Acceptance Subscale, CBI‐A = Cancer Behavior Inventory – Accepting Cancer/Maintaining Positive Attitude Subscale, AIS = Acceptance of Illness Scale, S‐PEACE = Single item peaceful acceptance of cancer, ICQ‐A = Illness Cognitions Questionnaire – Acceptance Subscale.	NS	NS	Acceptance of cancer was more strongly correlated with depressive and anxiety symptoms among cancer patients with early‐stage disease.	Acceptance was more strongly correlated with depressive and anxiety symptoms among cancer patients with early‐stage disease	Small‐to‐moderate, negative, and significant relationships were found between acceptance of cancer and general distress; cancer‐specific distress; depressive symptoms; and anxiety symptoms. Age, marital status, and stage of cancer were identified as significant moderators.	X
40	E. Secinti, W. Wu, E. F. Krueger, A. T. Hirsh, A. M. Torke, N. H. Hanna, N. Adra, G. A. Durm, L. Einhorn, R. Pili et al	2022	Acceptance refers to coming to terms with the situation or achieving a sense of peace. It is an active willingness to be present with cancer‐related realities while giving up efforts to judge or control cancer‐related appraisals or feelings.	Cancer, not specified	Peaceful Acceptance subscale of the PEACE measure.	NS	G 16.91 (SD = 3.29)	NS	All advanced patients.	NS	Perceived injustice was directly associated with psycho‐spiritual outcomes and indirectly related to these outcomes through acceptance. Findings were similar across advanced lung and prostate cancer patients.
41	S. T. Tang, W. C. Chang, J. S. Chen, W. C. Chou, C. H. Hsieh, C. H. Chen	2016	Emotional acceptance	Poor prognosis	Degree of acceptance: 1 (not at all accepted) to 7 (complete acceptance). High acceptance > 5, low acceptance < 5	Longitudinally from a year to 30 days before passing.	N Prognostic awareness with high acceptance: 43.3% to 32.5% closer to death. Prognostic awareness with low acceptance: 37.5% to 43.2	As death approached, accurate prognostic awareness increased (58.5% to 69.8%), prognostic acceptance decreased (43.4% to 32.5%).	NS	Participants who knew and highly accepted their prognosis were significantly less likely to experience severe anxiety symptoms than those who were unaware of or knew their prognosis but had difficulty accepting it.	NS
42	G. N. Thompson, H. M. Chochinov, K. G. Wilson, C. J. McPherson, S. Chary, F. M. O'Shea, D. R. Kuhl, R. L. Fainsinger, P. R. Gagnon, K. A. Macmillan	2009	NS	Not specified	One item on the SICS. “Do you feel that you can accept your situation and come to terms with all that is happening?”Acceptors if they scored 0‐2, non acceptors with 3 or above	NS	N 74.3% fully accepted the situation and have come to terms with it. 17.1% minimal to mild difficulty with accepting. 7.8% did not accept.	Less acceptance when: younger, higher education ans smaller social network. The predictors of acceptance were age, hopelessness, and communication issues.	Only metastatic cancer.	Nearly half (45.5%) the participants with acceptance difficulties met diagnostic criteria for a depressive or anxiety disorder compared with only 22.4% of those classified as Acceptors. Participants identified as Nonacceptors reported having significantly more Symptoms and Concerns.	NS
43	H. Wang, X. Sun, H. Yue, Y. Yang, D. Feng	2022	Acceptance of illness is defined as ‘approval of the fact of patients being ill, recognition of the need to adapt to the illness, and perception of addressing aversive consequences'.	Illness	Acceptance of illness scale (AIS)	NS	G 23.22 (6.90)	NS	All metastatic patients	NS	Patients' neuroticism, and extraversion were associated with their depression through the mediating effect of their own acceptance of illness (actor–actor effect). Patients' and partners' neuroticism and extraversion were associated with their own acceptance of illness, which in turn were negatively associated with the depression of their counterpart (actor–partner effect).
44	S. Wieder‐Huszla, J. Owsianowska, A. Chudecka‐Głaz, D. Branecka‐Woźniak, A. Jurczak	2022	NS	Illness	Acceptance of illness scale (AIS)	NS	G 26.65 (8.85)	The AIS scores correlated significantly and positively with the intensity of the constructive style of mental adaptation, and negatively with the intensity of the destructive style.	NS	NS	NS
45	R. Piotrkowska, A. Kruk, A. Krzemińska, W. Mędrzycka‐Dąbrowska, K. Kwiecień‐Jaguś	2023	Acceptance, which means “accepting a judgement, opinion, point of view or way of conduct, having favourable attitudes, or giving consent to something”.	Illness	Acceptance of Illness Scale (AIS)	NS	G 21.6 (SD = 7.32)	Pain, fatigue and diarrhoea decreased the acceptance of illness. Social and demographic factors did not influence acceptance.	NS	The greater acceptance of illness, the greater satisfaction with life in patients with cancer.	NS
46	S. Q. Chen, J. E. Liu, Z. Li, Y. L. Su	2017	Acceptance is a developing process that carries emotional and behavioral responses and has both passive and positive states.	Illness	Semistructured interview	NS	Patients developed an increasing acceptance of their breast cancer as their treatment stage progressed with time. This process was found to include five stages: non‐acceptance, passive acceptance, willingness to accept, behavioral acceptance, and transcendence of acceptance.	The extent to which the women with breast cancer accepted having the disease was found to increase with the treatment stage and as their treatment stage progressed with time.	NS	NS	NS
47	S. Fawson, Z. Moon, K. Novogrudsky, F. Moxham, K. Forster, I. Tribe, R. Moss‐Morris, C. Johnson, L. D. Hughes	2024	Acceptance is the awareness and willingness to experience private thoughts, feelings and emotions without trying to change or control them.	Acceptance (not specified), self‐acceptance, emotional acceptance and pain acceptance	Various measures, not specified.	NS	NS	NS	NS	The pooled correlation between acceptance and distress was significant, although with a small effect (rpooled = −0.24, 95% CI −0.34, −0.15, k = 16). Analyses revealed no significant moderators for the relationship between acceptance and distress. This suggests the relationships between acceptanceand distress did not differ for different stages of diagnosis, age, gender or time since diagnosis.	NS
48	D. Krok, E. Telka, D. Kocur	2024	NS	Adaptation to the disease in terms of the ability to accept health conditions and maintain overall life satisfaction.	Acceptance of Life with the Disease Scale	NS	G Mean 3.38, SD 0.71	Our results supported the mediational model in which meaning‐making and fear of recurrence serially mediated the relationship of both perceived and received social support with illness acceptance.	NS	NS	NS
49	D. Krok. E. Telka, M. Moron	2024	NS	Adaptation to illness, understood as an individual's ability to accept health conditions in the context of their own illness.	Acceptance of Life with the Disease Scale	NS	NS	Higher secure attachment and low insecure attachment scores were associated with a higher sense of coherence and better illness acceptance both in women and partners. Results of actor–partner interdependence mediation models indicated that most associations between attachment styles and illness acceptance were mediated by sense of coherence within both intrapersonal (actor–actor) and interpersonal (actor–partner) effects.	NS	NS	NS
50	A. Królikowska, M. Stanisławska, M. Starczewska, A. Rybicka, K. Rachubinska	2024	NS	The patient's level of coming to terms with the disease.	Acceptance of Illness Scale (AIS)	NS	G Mean 29.68	Accepatance lower in older patients, patients who had traditional surgery and those after cystectomy. Accepatance was higher in patients with vocational and secondary education, residents of medium‐sized and large cities, and among patients hospitalized once, as well as patients not treated with chemotherapy. Acceptance higher in patients with higher fighting spirit as well as higher constructive style.	NS	NS	NS
51	M. Matbouei, M. Samsami, M. Soleimani	2024	The definition of acceptance in cancer recurrence includes the patient's attitude and behavior to maintain quality of life.	Cancer recurrence	Semistructured interview	NS	NS	The patient's psychological preparation, support systems, behavior of healthcare providers, and rebuilding trust are the determining factors in acceptance of recurrence.	NS	NS	NS
52	C. Sauer, T. Hansen, H. G. Prigerson, J. W. Mack, T. J. Bugaj, G. Weißflog	2024	The ‘active willingness to be present with cancer‐related realities while giving up efforts to judge or control cancer‐related appraisals or feelings. In addition, cancer acceptance involves a behavioral willingness in response to cancer‐related stressors, resulting in actions aligned with deeply held values.’	Cancer‐related realities and cancer‐related stressors	Peace, Equanimity, and Acceptance in the Cancer Experience (PEACE) questionnaire	NS	NS	Patients on sick leave reported significantly lower peaceful acceptance than patients not on sick leave. Peaceful acceptance correlated significantly with age.	NS	Peaseful acceptance showed negative associations with depression, anxiety, distress, psychological inflexibility, and positive associations with HRQoL, acceptance, resilience, and mindfulness.	NS
53	F. Uslu‑Sahan, N. Gulcan	2024	Acceptance of disease, which may be defined as recognizing the need to adjust to the condition, tolerating its unexpected and uncontrollable manifestations, and deciding to cope with the bad effects.	Illness	Acceptance of Illness Scale (AIS)	NS	G, Mean 23.94 ± 5.81	The acceptance of illness level was higher in the participants aged 65 and over, unemployed, had an income equal to/higher than expenses, had a cancer early‐stage, the onset of diagnosis over 12 months, and had a type of treatment single. Acceptance of illness was negatively related to supportive care needs.	NS	NS	NS
54	B. Xu, M. Qian, L. Zhu, Z. Lu	2024	A positive attitude: patients freely embrace their state of health and their surroundings and constructively react to stressful circumstances by adopting an accepting and entrepreneurial coping strategy.	Not specified	Positive Acceptance Scale	NS	G, Mean/SD: 49.20 ± 4.88	Uncertainty tolerance was negatively correlated with positive acceptance.	NS	NS	NS

*Note:* NS = not specified.

**TABLE B2:** Definition, focus, measurement.

No.	Authors	Definition of acceptance	Focus of acceptance	Measurement of acceptance
1	J. Hinton	Acceptance was described in terms of death's inevitability, faith and spiritual values, life's diminishing rewards (lack of pleasure), completing life, final benefits, humour, sharing, final activities etc. Individuals often used more than one concept. It is explained as coming to terms with this prospect – or the opposite.	Death	Patients and relatives were interviewed separately each week for eight weeks and fortnightly from then on. Acceptance rating 1–9 by researcher.
4	A. Czerw, U. Religioni, A. Deptata	Acceptance of illness is an emotional determinant of functioning with and adaptation to the illness and is manifested in a small intensity of negative emotions associated with the illness.	Illness	Acceptance of Illness Scale (AIS)
6	A. Nowicki, P. Sianoszek, P. Farbricka	Acceptance allows for a rational assessment of a difficult situation in which a patient is currently ill, which makes it possible to have a real impact on his/her health by taking up the fight against the disease.	Illness	Acceptance of Illness Scale (AIS)
7	A. Ray, S. D. Block, R. J. Friedlander, B. Zhang, P. K. Maciejewski, H. G. Prigerson	Acceptance is finding a deep sense of peace or harmony	Not specified	NIA/Fetzer Multidimensional Measure of Religiousness/Spirituality
8	A. Czerw, M. Bilinska, A. Deptata	The assessment of acceptance of illness is conducted at two levels: emotional and cognitive‐behavioural.	Illness	Acceptance of Illness Scale (AIS)
11	C. E. Mosher, E. Krueger, E. Secinti, S. A. Johns	Acceptance is full awareness of experiences without defense, and defusion involves noticing thoughts without getting caught up in their content.	Cancer, not specified	Peaceful Acceptance subscale of the Peace, Equanimity, and Acceptance in the Cancer Experience (PEACE)
12	C. F. Pereira, K. Cheung, E. Alie, J. Wong, M. J. Esplen, Y. W. Leung	In the context of a terminal illness, acceptance is considered to be an active approach in which a person does not attempt to judge or change the course of their illness or their internal experience	Illness, cancer, death	Directed content analysis of transcripts
17	K. Chinh, E. Secinti, S. A. Johns, A. T. Hirsh, K. D. Miller, B. Schneider et al.	Illness acceptance is defined as a sense of peace in confronting mortality and the losses associated with the illness. This concept suggests that accepting the reality of a chronic or terminal illness can lead to better psychological outcomes for patients by fostering a more peaceful and less resistant mindset towards their condition.	Cancer, not specified	Peace, Equanimity, and Acceptance in the Cancer Experience measure (PEACE)
18	E. Cipora, M. Konieczny, J. Sobieszczański	Acceptance of illness is coming to terms with own state of health, it enables the actual assessment of one's own health situation, and often provides incentives to struggle against the disease.	Illness	Acceptance of Illness Scale (AIS)
19	T. N. Elumelu, C. C. Asuzu, E. O. Akin‐Odanye	Acceptance of a chronic health condition is a coping strategy that includes both direct action and passive components. Acceptance is defined as acknowledging the reality of the illness and learning to live with it, rather than resigning to fate. It involves recognizing that medical or behavioral therapies may not eradicate the health condition entirely and shifting focus from pain and other symptoms to other aspects of life.	Not specified	Brief Cope questionnaire
21	K. Suwannapong, S. Thanasilp, D. L. Doutrich, L. Akkayagorn, N. H. Long, J. Chimluang, N. Pudtong, R. Upasen	Thai Buddhism encourages people to think of their death as a natural process and be aware that they must die someday; to understand it is impossible for them to go beyond death.	Death	Buddhist Death Acceptance Scale
23	D. Krok, E. Telka, M. Moroń	Coming to terms with one's own state of health	Disease	The Acceptance of Life with the Disease
24	D. Krok, E. Telka, B. Zarzycka	One's adaptation to the disease in terms of the ability to accept health conditions and maintain overall life satisfaction.	Disease	The Acceptance of Life with the Disease
25	K. Kuba, G. Weißflog, H. Götze, F. García‐Torres, A. Mehnert, P. Esser	Acceptance is psychological flexibility. Acceptance is defined by staying in contact with the present moment, which means to accept any emotional, cognitive or physical experiences without regulating or controlling them. Furthermore, it entails commitment, which means that behavior persists or is changed in order to pursue personal values and goals.	Not specified	Acceptance and Action Questionnaire (AAQ‐II)
26	J. W. Mack, M. Nilsson, T. Balboni, R. J. Friedlander, S. D. Block, E. Trice, H. G. Prigerson	The patient's sense of acceptance, calmness, and peace. The emotional experience of coming to accept one's terminal illness.	Cancer, not specified	Peace, Equanimity, and Acceptance in the Cancer Experience (PEACE)
27	S. L. Manne, D. A. Kashy, S. Virtue, K. R. Criswell, D. W. Kissane, M. Ozga, C. J. Heckman, J. Stapleton, L. Rodriguez	Acceptance consists of accepting that the event has occurred as well as emotional acceptance, which is a willingness to feel one's positive and negative emotions without trying to control, change, or reject these feelings.	Not specified	Acceptance subscale of the COPE.
28	T. V. Merluzzi, N. Salamanca‐Balen, E. J. Philip, J. M. Salsman	Religious and spiritual processes	Illness, not specified	Meaning and Peace subscales of the FACIT‐sp
29	M. Pasek, G. Dębska, E. Wojtyna	Acceptance of illness, which can be defined as approval of the fact of being ill, realising the need to adapt to the condition, tolerance to its unexpected and uncontrolled manifestations, and a decision to cope with unfavourable consequences of experienced ailments.	Illness	Acceptance of Illness Scale (AIS)
30	R. Philipp, A. Mehnert, C. Lo, V. Müller, M. Reck, S. Vehling	Death acceptance refers to the absence of fear and anxiety about death and the acceptance of death as a natural aspect of life.	Death	Death acceptance subscale of the Life Attitude Profile‐Revised (LAP‐R)
31	M. Pinquart, C. Fröhlich, R. K. Silbereisen, U. Wedding	In the present study, death acceptance is defined as a calm attitude toward the finiteness of one's life, and seeing death as an unchangeable fact of life that also might have some positive aspects	Death	Five item likertscale by Brandstadter
33	R. M. Quinto, F. De Vincenzo, L. Campitiello, M. Innamorati, E. Secinti, L. Iani	An active willingness to be present with cancer‐related realities while giving up efforts to judge or control cancer‐related appraisals or feelings.	Not specified, illness	Acceptance subscale of the BriefCOPE (B‐COPE), Acceptance of Disability Scale‐Revised, Acceptance of Illness Scale (AIS)
34	R. Upasen, S. Thanasilp, L. Akkayagorn, J. Chimluang, W. Tantitrakul, D. L. Doutrich, W. Saengpanya	Death acceptance is a dynamic and iterative process that involves three phases, namely, engaging suffering, being open‐minded about death, and adhering to Buddhist practices for increasing death consciousness.	Death	Interview
36	U. Religioni, A. Czerw, A. Deptała	Acceptance of disease is simultaneously conducted at two levels: the emotional and cognitive‐behavioral one. Patients learn to accept not only the symptoms but also the resulting changes in the quality of life, limitation of self‐reliance and independence, and thus the change of their individual roles in their families and the society.	Illness	Acceptance of illness scale (AIS)
38	E. Sachs, E. Kolva, H. Pessin, B. Rosenfeld, W. Breitbart	Acceptance implies a realistic understanding of prognosis and a willing relinquishing of inflexible or unrealistic hopes.	Death, poor prognosis	Semi‐structured interview
39	E. Secinti, D. B. Tometich, S. A. Johns, C. E. Mosher	Broadly, we conceptualize acceptance of cancer as an active willingness to be present with cancer‐related realities while giving up efforts to judge or control cancer‐related appraisals or feelings. In addition, acceptance of cancer involves a behavioral willingness in response to cancer‐related stressors, resulting in action aligned with deeply held values.	Not specified, illness, cancer	BCOPE‐A = Brief COPE – Acceptance Subscale; COPE‐A = COPE Acceptance Subscale, CBI‐A = Cancer Behavior Inventory – Accepting Cancer/Maintaining Positive Attitude Subscale, AIS = Acceptance of Illness Scale, S‐PEACE = Single item peaceful acceptance of cancer, ICQ‐A = Illness Cognitions Questionnaire – Acceptance Subscale.
40	E. Secinti, W. Wu, E. F. Krueger, A. T. Hirsh, A. M. Torke, N. H. Hanna, N. Adra, G. A. Durm, L. Einhorn, R. Pili et al	Acceptance refers to coming to terms with the situation or achieving a sense of peace. It is an active willingness to be present with cancer‐related realities while giving up efforts to judge or control cancer‐related appraisals or feelings.	Cancer, not specified	Peaceful Acceptance subscale of the PEACE measure.
41	S. T. Tang, W. C. Chang, J. S. Chen, W. C. Chou, C. H. Hsieh, C. H. Chen	Emotional acceptance	Poor prognosis	Degree of acceptance: 1 (not at all accepted) to 7 (complete acceptance). High acceptance > 5, low acceptance < 5
43	H. Wang, X. Sun, H. Yue, Y. Yang, D. Feng	Acceptance of illness is defined as ‘approval of the fact of patients being ill, recognition of the need to adapt to the illness, and perception of addressing aversive consequences'.	Illness	Acceptance of illness scale (AIS)
45	R. Piotrkowska, A. Kruk, A. Krzemińska, W. Mędrzycka‐Dąbrowska, K. Kwiecień‐Jaguś	Acceptance, which means “accepting a judgement, opinion, point of view or way of conduct, having favourable attitudes, or giving consent to something”.	Illness	Acceptance of Illness Scale (AIS)
46	S. Q. Chen, J. E. Liu, Z. Li, Y. L. Su	Acceptance is a developing process that carries emotional and behavioral responses and has both passive and positive states.	Illness	Semistructured interview
47	S. Fawson, Z. Moon, K. Novogrudsky, F. Moxham, K. Forster, I. Tribe, R. Moss‐Morris, C. Johnson, L. D. Hughes	Acceptance is the awareness and willingness to experience private thoughts, feelings and emotions without trying to change or control them.	Acceptance (not specified), self‐acceptance, emotional acceptance and pain acceptance	Various measures, not specified.
51	M. Matbouei, M. Samsami, M. Soleimani	The definition of acceptance in cancer recurrence includes the patient's attitude and behavior to maintain quality of life.	Cancer recurrence	Semistructured interview
52	C. Sauer, T. Hansen, H. G. Prigerson, J. W. Mack, T. J. Bugaj, G. Weißflog	The ‘active willingness to be present with cancer‐related realities while giving up efforts to judge or control cancer‐related appraisals or feelings. In addition, cancer acceptance involves a behavioral willingness in response to cancer‐related stressors, resulting in actions aligned with deeply held values.’	Cancer‐related realities and cancer‐related stressors	Peace, Equanimity, and Acceptance in the Cancer Experience (PEACE) questionnaire
53	F. Uslu‑Sahan, N. Gulcan	Acceptance of disease, which may be defined as recognizing the need to adjust to the condition, tolerating its unexpected and uncontrollable manifestations, and deciding to cope with the bad effects.	Illness	Acceptance of Illness Scale (AIS)
54	B. Xu, M. Qian, L. Zhu, Z. Lu	A positive attitude: patients freely embrace their state of health and their surroundings and constructively react to stressful circumstances by adopting an accepting and entrepreneurial coping strategy.	Not specified	Positive Acceptance Scale
